# Emergent Locomotion in Self‐Sustained, Mechanically Connected Soft Matter Rings

**DOI:** 10.1002/adma.202503519

**Published:** 2025-04-30

**Authors:** Hongshuang Guo, Kai Li, Arri Priimagi, Hao Zeng

**Affiliations:** ^1^ Faculty of Engineering and Natural Sciences Tampere University P.O. Box 541 Tampere FI‐33101 Finland; ^2^ Department of Civil Engineering Anhui Jianzhu University Hefei 230601 China

**Keywords:** collective effect, liquid crystal elastomers, mechanical coupling, non‐equilibrium, self‐sustained

## Abstract

In nature, the interplay between individual organisms often leads to the emergence of complex belabours, of which sophistication has been refined through millions of years of evolution. Synthetic materials research has focused on mimicking the natural complexity, e.g., by harnessing non‐equilibrium states to drive self‐assembly processes. However, it is highly challenging to understand the interaction dynamics between non‐equilibrium entities and to obtain collective behavior that can arise autonomously through interaction. In this study, thermally fueled, twisted rings exhibiting self‐sustained movements are used as fundamental units and their interactive behaviors and emergent functions are investigated. The rings are fabricated from connected thermoresponsive liquid crystal elastomers (LCEs) strips that undergo zero‐elastic‐energy‐mode, autonomous motions upon a heat gradient. Single‐ring structures with various twisting numbers and nontrivial links, and connected knots where several LCE rings (*N* = 2,3,4,5) are studied and linked. The observations uncover that controlled locomotion of the structures can emerge when *N* ≥ 3. The locomotion can be programmed by controlling the handedness at the connection points between the individual rings. These findings illustrate how group activity emerges from individual responsive material components through mechanical coupling, offering a model for programming autonomous locomotion in soft matter constructs.

## Graphical abstract

Thermally fueled, self‐moving twisted rings made from liquid crystal elastomers are investigated. By studying single rings and linked knots, it is shown how autonomous locomotion emerges through coupling between the rings. The movement is programmable via handedness control at connection points, offering insights into collective behavior and programmable motion in soft materials.

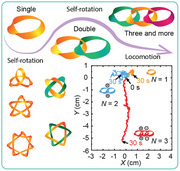

## 1 Introduction

Over the past decades, bioinspired materials research has topically evolved from structural mimics and chemical bionics towards materials with life‐like functions.^[^ ^1^, ^2^, ^3^, ^4^ ^]^ Such functions include, e.g., perception,^[^ ^5^ ^]^ adaptation,^[^ ^6^, ^7^ ^]^ and self‐regulation,^[^ ^8^ ^]^ and alike in biological systems, they necessitate the material to be driven out of equilibrium. In this context, stimuli‐responsive materials fueled by external energy sources such as light, heat, humidity, or chemical substances, are of particular importance.^[^ ^9^ ^]^ Even relatively simple synthetic materials can be programmed to undergo feedback‐driven motions or self‐regulating actions providing simplistic mimics to complex biological processes such as homeostasis^[^ ^10^, ^11^ ^]^ or environmental sensing and response.^[^ ^12^ ^]^ Such non‐equilibrium processes provide great potential in fields such as micro‐robotics, biomedical engineering, and smart textiles and wearables. However, contemporary research primarily focuses on the performance of individual objects.^[^ ^13^, ^14^, ^15^, ^16^ ^]^ It is pertinent yet highly challenging to move from individuals towards collectives with emergent properties that require interaction between individual object.

Interactive behavior and different forms of communication are prevalent in natural systems.^[^ ^17^, ^18^ ^]^ This is evident across various biological scales: bacteria work together to form biofilms,^[^ ^19^ ^]^ locusts march in swarms,^[^ ^20^ ^]^ termites build enormous and intricate structures,^[^ ^21^ ^]^ and complex swarming activities.^[^ ^22^, ^23^ ^]^ One of the key characteristics of these interactive systems is self‐organization,^[^ ^24^ ^],^ i.e., the shape and group velocity of the ant cluster are reconfigured based on mechanical interaction within the network. In other words, the individuals dictate the properties and actions of the entire group, an impossible feat for individual elements or entities that do not mechanically interact. In synthetic materials research, examples include the development of self‐assembling colloidal systems,^[^ ^25^ ^]^ magnetically controlled soft robots,^[^ ^26^ ^]^ responsive polymer networks,^[^ ^27^ ^]^ and programmable matter that can change shape or properties in response to external stimuli.^[^ ^28^ ^]^ However, the interaction between non‐equilibrium, locomotive structures, or a facile strategy for programming such interactive behavior, is yet to be demonstrated.

To attain controllable collective behavior, natural design principles suggest a decentralized control strategy without a pre‐designed overall stimulus pattern to drive the group action.^[^ ^29^ ^]^ In this case, a leader is absent and interactions between individuals and their immediate surroundings induce sophisticated group patterns. Interactions have been achieved in inanimate responsive material systems, for instance, synchronization via mechanical coupling between self‐oscillating cantilevers,^[^ ^27^ ^]^ and the appearance of collective effects within active elements^[^ ^28^ ^]^ upon the stimulation of modulated fields.^[^ ^30^ ^]^ Intriguing research questions thus arise: Can non‐equilibrium material pieces exhibiting self‐sustained motions interact with each other mechanically? If yes, will the interaction give rise to novel locomotive behaviors? If yes, can such activities be pre‐designed and programmed?

In this study, we try to answer the above questions by investigating how the mechanical coupling between individual synthetic material objects can influence the locomotion of the whole group. The experiment is made by using thermally driven rings undergoing self‐sustained motions as the fundamental interacting units. We demonstrate various self‐rotation modes in single rings and explore the interactions between multiple rings within diverse kinds of closely connected loops. By controlling only the handedness at the connecting points between neighboring rings, the proposed model system provides the programmability to obtain versatile locomotion of dissipative soft structures on a two‐dimensional, heat‐fueled plane.

## 2 Results and Discussion

**Figure 1:**
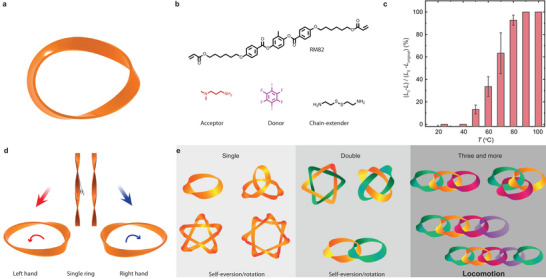
System concept. a) Schematic picture of a twisted ring knot. b) Chemical structures of the molecules used in LCE synthesis. c) Heat‐induced deformation of the LCE. *L_0_* stands for the initial length (after stretching) and *L* is the contracted length under heating. *L_original_* is the initial length before stretching. d) The diagram includes the twisted strips and rings of left‐handed and right‐handed helices, along with the rotation direction of the rings under heating. e) A schematic diagram illustrating the structures of a single ring, two connected rings, and three or more connected rings. The modes of movement primarily include self‐rotation and translation.

The mechanically self‐sustained strefoilystem is based on a ring structure made of a twisted soft material strip, as shown in **Figure** **1**a. This closed ring, with a minimal half cycle of twisting, provides zero elastic energy mode (ZEEM) mechanics^[^ ^31^ ^]^ for continuous eversion upon thermal gradient by placing it on top of a hot stage. ZEEM^[^ ^31^ ^]^ is a mechanical structure in which the potential elastic energy associated with deformation is minimized. A typical example is a torus structure that can perform inversion or eversion with arbitrary rotating angles. A closed strip ring is not a ZEEM, however, with a minimal half cycle of twisting, a closed ring turns into ZEEM.^[^ ^32^, ^33^ ^]^ It exhibits ZEEM‐based continuous eversion upon thermal gradient by placing it on top of a hot stage. In brief, the ZEEM working principle is to synergistically utilize a pre‐strain field (due to the closed knot structure) and an orthogonal heat gradient field (due to the hot plate) within a closed ring, to bring about a driving torque for eversion. When the power of the torque subdues the friction and material internal losses, a continuous version emerges. ZEEM has been utilized for applications in soft robotics and artificial muscles by using a wide range of responsive materials.^[^ ^5^, ^32^, ^33^, ^34^, ^35^, ^36^ ^]^ The ring used in this study is made of liquid crystal elastomer (LCE),^[^ ^37^ ^]^ with chemical composition depicted in Figure 1b. The introduction of dynamic halogen bonds introduced as supramolecular crosslinks allows for post‐programming of the strip structure and deformation, which can eliminate Michell's instability caused by the stress generated from twisting during bonding.^[^ ^38^ ^]^ The LCE is synthesized via Aza–Michael addition reaction to produce a polydomain‐oriented polymer,^[^ ^39^, ^40^ ^]^ followed by stretching of the strips to attain monodomain orientation that endows thermo‐responsive uniaxial contraction of ca. 50% upon heating to 90 °C (Figure 1c; Figure S1a,b, Supporting Information). The order parameter is estimated to be around 0.7 based on Wide‐angle X‐ray scattering (WAXS) data (Figure S2, Supporting Information), and is gradually lost upon heating (Figure S3a–c, Supporting Information). The glass transition temperature (*T_g_*) of the LCE is −3 °C (Figure S4a, Supporting Information) and it has a Young's modulus of *ca*. 3 MPa (Figure S4b, Supporting Information).

The utilization of an external force field after the polymerization provides a valuable tool for programming the topology of ZEEM rings (Figure S5, Supporting Information). By varying the twisting strength one can produce different twisting numbers, *N*_t_, and helical angles, *θ*_f_, the angle between helical thread and radial direction schematized in Figure 1d. Different *N*_t_ result in different autonomous rolling speeds on a hotplate (Figure S6, Supporting Information). After connecting the two ends of the strip into a closed ring, the twisted surface produces anisotropic friction at the thread–ground interface during eversion, which introduces angular momentum of the ring and causes unidirectional rotation of the structure. Under steady movement, the angular velocity of eversion *ω*_Ε_, is entrained with the angular velocity of rotation *ω*_Ρ_ (modeling in Supporting Information). The latter is controlled by the handedness of the structure as determined by the sign of *θ*_f_. The rotation direction thus depends on the twisting direction, as indicated by the schematic drawing in Figure 1d.

Figure 1e shows the roadmap adopted in this study to develop mechanically interacting rings. It starts by making single closed rings with different *N*_t_. The complexity of a single ring is further increased by using diverse designs with nontrivial linkages. Then we move to make structures comprising several (*N*) connected rings. In the following, we will show that the complex configurations in the single and dual rings allow diverse self‐sustained rotation with the possibility of the random walk but no possibility of net displacement of the center of the mass. For *N* ≥ 3, programmable locomotion emerges.

**Figure 2:**
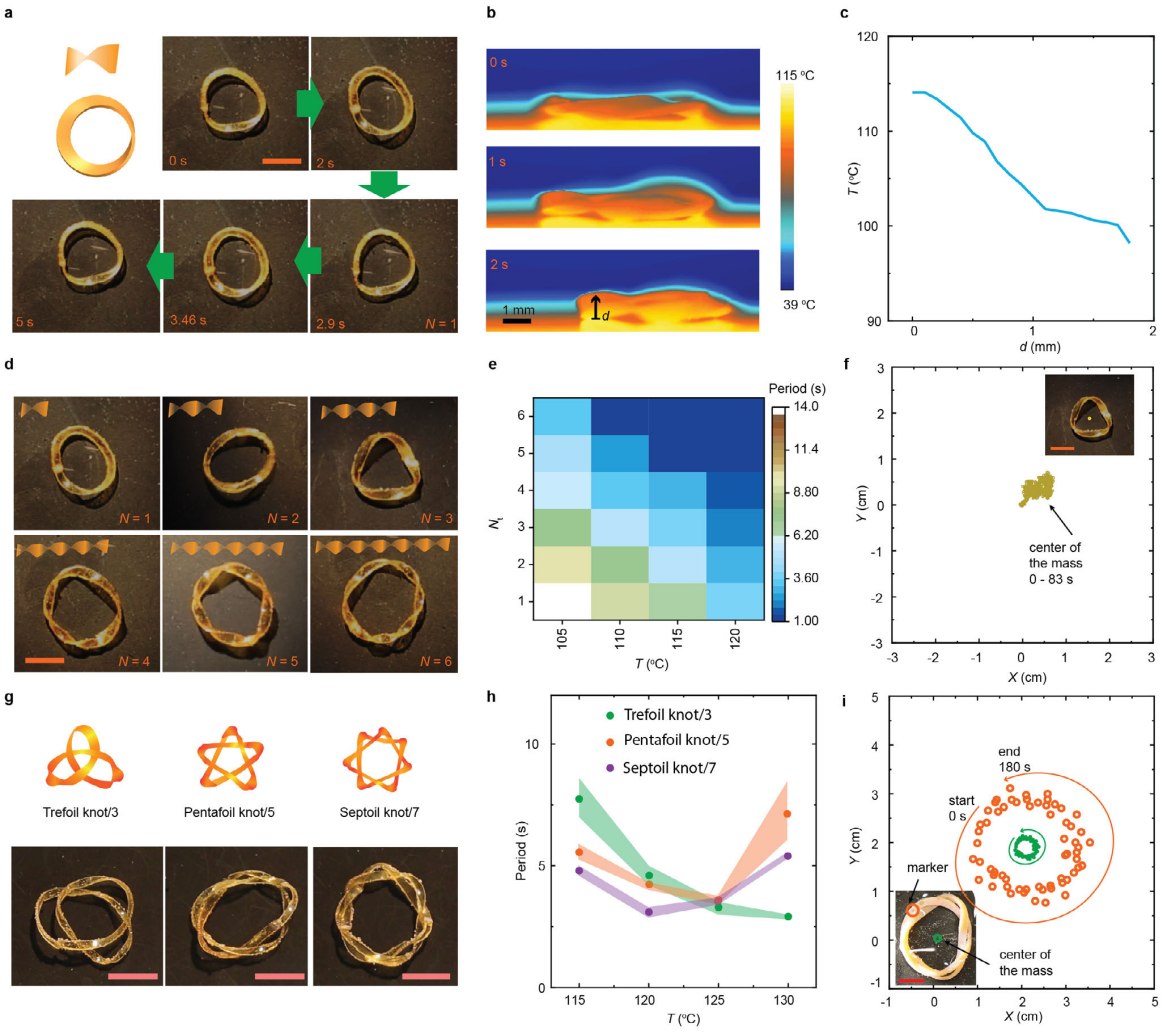
Basic characteristics of a single ring. a) Snapshots of a ring with one twist rotating on a 115 °C hot plate. b) Infrared images of the self‐rotating ring. c) The thermal gradient across the width is deduced from (b). d) Photographs of strip rings with different twist numbers. e) Color map summarizing the eversion period as a function of temperature and twist number. f) The displacement of the center of mass in a single ring (*N*_t_ = 3). The inset depicts the position of the center of mass. g) Photographs of single rings in the form of a Trefoil Knot/3, Pentafoil Knot/5, and Septoil Knot/7 and h) their change in eversion period upon heating. Error bars indicate standard deviation for *n* = 3 measurements. The same sample was measured repeatedly. i) The movement trajectory and mass center for the Pentafoil Knot/5. The inset depicts the positions of the tracked points. The time interval is 3 seconds. All scale bars are 1 cm.

We fabricated LCE strips with different *N*_t_ and glued their ends to create the twisted ring structures (**Figure** **2**a–d). On a hot plate, there exists a temperature gradient from 115 °C (bottom surface of the ring) to 95 °C (top surface of the ring), as indicated by the infrared camera images shown in Figure 2b,c. At the same time, the overall diameter of the ring shrinks (Figure S7, Supporting Information). Then, the ring starts to evert, exhibiting continuous, ZEEM‐driven motion. The eversion speed depends on the ring dimensions, as demonstrated in Figure S8 (Supporting Information). Fixing the diameter, *N*_t_, and temperature, narrower rings rotate faster (Figure S8a, Supporting Information). By increasing *N*_t_ and temperature, the rings with the same width exhibit a quicker eversion. The mapping of *N*_t_, temperature and eversion speed is shown in Figure 2e, with more details in Figure S9 and Video S1 (Supporting Information). The tracking of the center of mass during the self‐rotation is shown in Figure 2f. Despite minor shifts due to the random fluctuation in substrate friction, the center of mass of a single ring remains largely intact.

It is important to note that the self‐sustained eversion of a twisted strip ring differs from that of a closed cylindrical ring (Figure S10a,b, Supporting Information). Cylindrical rings exhibit eversion without clockwise or anticlockwise rotation,^[^ ^31^, ^36^ ^]^ and it returns to its initial position right after completing one eversion cycle. In contrast, in a twisted strip ring, the surface position shifts with a specific angle after one eversion period, due to the friction anisotropy.

The mathematics of topological geometry provides inspiration for developing closed rings with enhanced complexity.^[^ ^41^ ^]^ We exemplify this by the Trefoil, Pentafoil, and Septoil knots as shown in Figure 2g and Video S2 (Supporting Information). *N*_c_ is denoted as the crossing points within the ring loop, as *N_c_* = 3, 5, and 7 for Trefoil, Pentafoil, and Septoil knots, respectively.^[^ ^42^ ^]^ Upon heating to 115–120 °C, the eversion speed increases with *N*_c_ (Figure 2h). At 130 °C, the Trefoil knot showed a further increase in speed. In contrast, both the Pentafoil and Septoil knots rotate slower when the temperature exceeds 125 °C. To understand this temperature‐dependent behaviors, we further study the influence of sample aspect ratio on the eversion behaviour. The results are shown in Figure S11 (Supporting Information). For the samples with high aspect ratio among all types of knots, i.e., Trefoil, Pentafoil and Septoil knots, the eversion speed trends to increase along the temperature. However, in the cases of Pentafoil and Septoil Knots (higher *N*_c_ compared to Trefoil knot), samples with low aspect ratio show a slowing down of eversion along the temperature. The above observations suggest that a competitive mechanism influences the motion. On one hand, the elevated temperature generally accelerates the deformation speed and thus reduces the eversion period. On the other hand, the heat induces shrinkage of knot diameter, as indicated in Figure S12 (Supporting Information) and visualized in Figure S11d–f (Supporting Information). At higher temperatures the distance between the two crossing points is reduced, as such, the strip becomes less freedom during the eversion/rotation. Accounting also for the fact that heat‐induced softening of LCE generally increases the friction coefficient,^[^ ^43^ ^]^ a ring with higher *N*_c_, and lower aspect ratio is expected to experience higher friction upon increasing temperature, thus slowing down the eversion speed. The competition between these mechanisms leads to the acceleration‐then‐deceleration of eversion, as exhibited in Figure 2h. The marked point on the ring surface exhibits rotary motion trajectory as shown in Figure 2i and Figure S13 (Supporting Information). Similar to a single ring, these more complex knots do not produce net displacement of the center of mass during the self‐sustained motion, as shown in the inset of Figure 2i.

**Figure 3:**
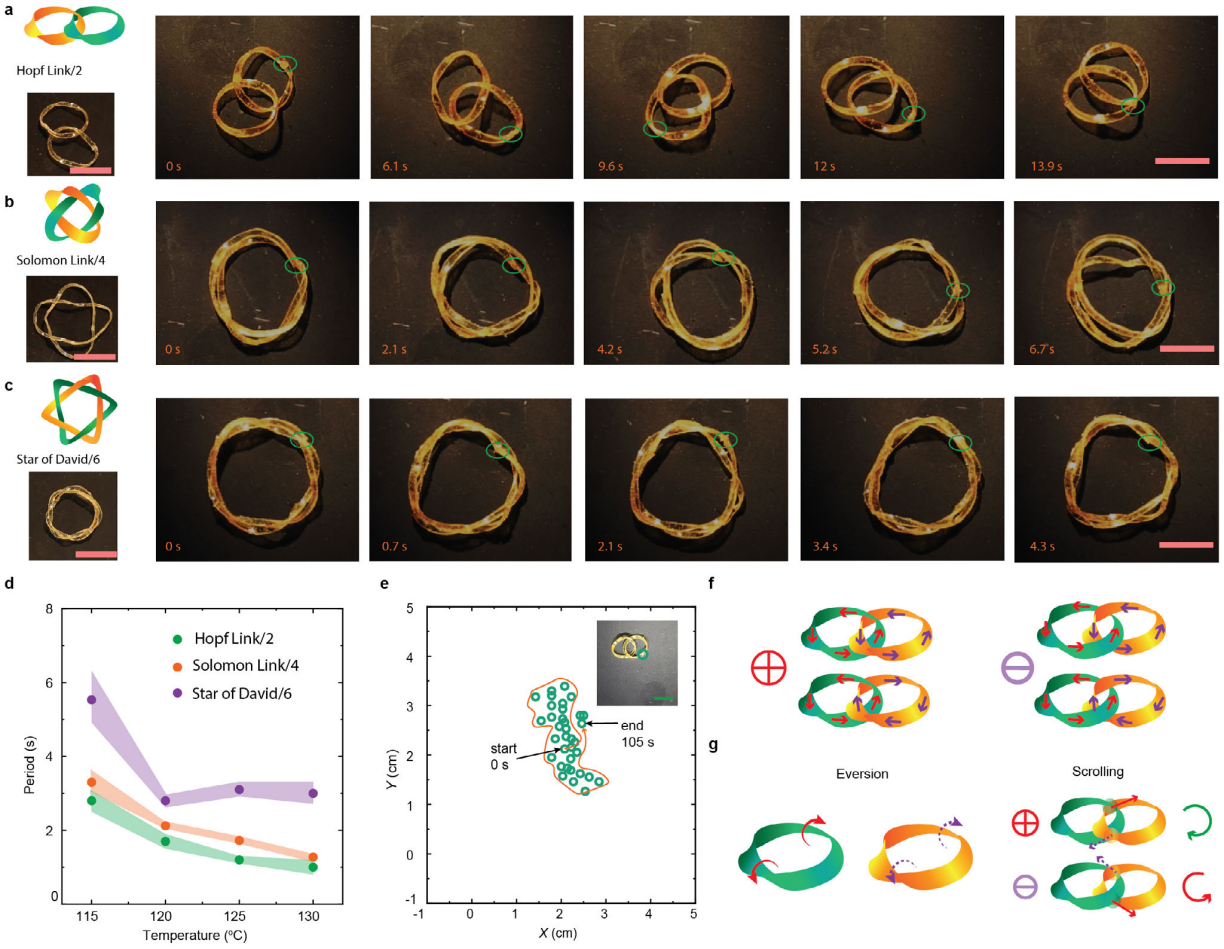
Motion patterns of two connected rings. a–c) Photographs of two linked rings in the form of a Hopf Link/2, Solomon Link/4, and Star of David/6 at different times during rotation. d) The change in eversion period with temperature for different types of linked rings. e) The movement trajectory of the Hopf Link/2 is viewed from above. The time interval is 3 seconds. f) The handiness definition method. When the connected ring pair placed horizontally, starting from the leftmost point and following the strip direction clockwise, if the left ring goes above the right one at the first crossing point, we define the connection as ⨁. If the left ring is placed below the right one, it is defined as ⊖. g) The kinetic analysis of both ⨁ and ⊖ connections. Error bars indicate standard deviation for *n* = 3 measurements. The same sample was measured repeatedly. All scale bars are 1 cm.

**Figure** **3**a–c show the self‐sustained motions of two interacting rings connecting into a closed loop in the form of Hopf Link/2, Solomon Link/4, and Star of David/6,^[^ ^44^ ^]^ where 2, 4, and 6 refer to the total crossing points in the link diagram. Upon heating, the eversion speed of the linked ring pairs decreases with the number of contact points, as shown in Figure 3d (see also Figure S14 and Video S3, Supporting Information). When increasing the temperature, the Hopf Link/2 and Solomon Link/4 rotate faster, while the eversion speed of Star of David/6 initially increases and then drops. We ascribe this to the same effect as with the single knot ring. High temperature induces shrinkage of knot diameter (Figure S15, Supporting Information), and the strip segment between two crossing points becomes less freedom for the eversion/rotation. An enhanced friction occurs between rings causing a reduced rotation upon high temperature. The reproducibility of different knots and the temperature‐dependent eversion speed trend are given in Figure S16 (Supporting Information), which shows consistency across different samples.

Based on Figure S17a–c (Supporting Information), the center‐of‐mass movements of the Solomon Link/4 and Star of David/6 are similar to a single ring. The trajectory of Hopf Link/2, instead, shows a complicated pattern (Figure 3e; Figure S18, Supporting Information). To study the interaction between two linked rings in more detail, we chose the Hopf Link/2 as an example, and defined the type of connection between the rings as follows (see Figure 3f for schematic drawing): if the connected ring pair is placed horizontally, and starting to follow the structure clockwise from the leftmost point, if the left ring goes above the right one at the first crossing point, we define the connection as ⨁. If the left ring is below the other ring at the first crossing point, such connection is defined as ⊖. We observed that regardless of the rotation direction of the individual rings, ⨁ connection always results in an entangled rotation in the clockwise direction. Conversely, ⊖ connection leads to a counter‐clockwise rotation. For further details of the movements of ring pairs, see Figure S19 (Supporting Information). We explain the emerged rotation handedness in the interacting ring pair as follows. During steady eversion, each ring continuously flips from inward to outward (eversion), alike a wheel scrolling on a ground surface to provide momentum for forward movement. With ⨁ connection, the left ring placed on top of the right one at the up‐contact point, scrolls along the strip direction of the right ring, yielding a clockwise crawling motion. The right ring is placed on top of the left one at the bottom‐contact point, creating a scrolling along strip direction with the same handedness. The eventual effect is that both rings get entangled, rotating around each other in the clockwise direction (see Figure 3g for kinetic analysis of the ⨁ and ⊖ connections). The same principle applies to ⊖ connection for generating group motion in an anticlockwise direction. The message conveyed by the above observations is twofold. First, the interaction between two rotating rings produces a torque that induces handedness in the group motion causing an increased displacement in their center of mass (Figure 3e; Video S4, Supporting Information) as compared to the single ring. Second, a change of ring connection can alter the group rotation direction, providing an opportunity for programming the movement of the connected rings independently from the rotation direction of the individual rings. In the following, we demonstrate emergent locomotion in closely connected rings with *N* = 3, with programmable directionality by the connection (⨁ or ⊖) between the ring pairs.

**Figure 4:**
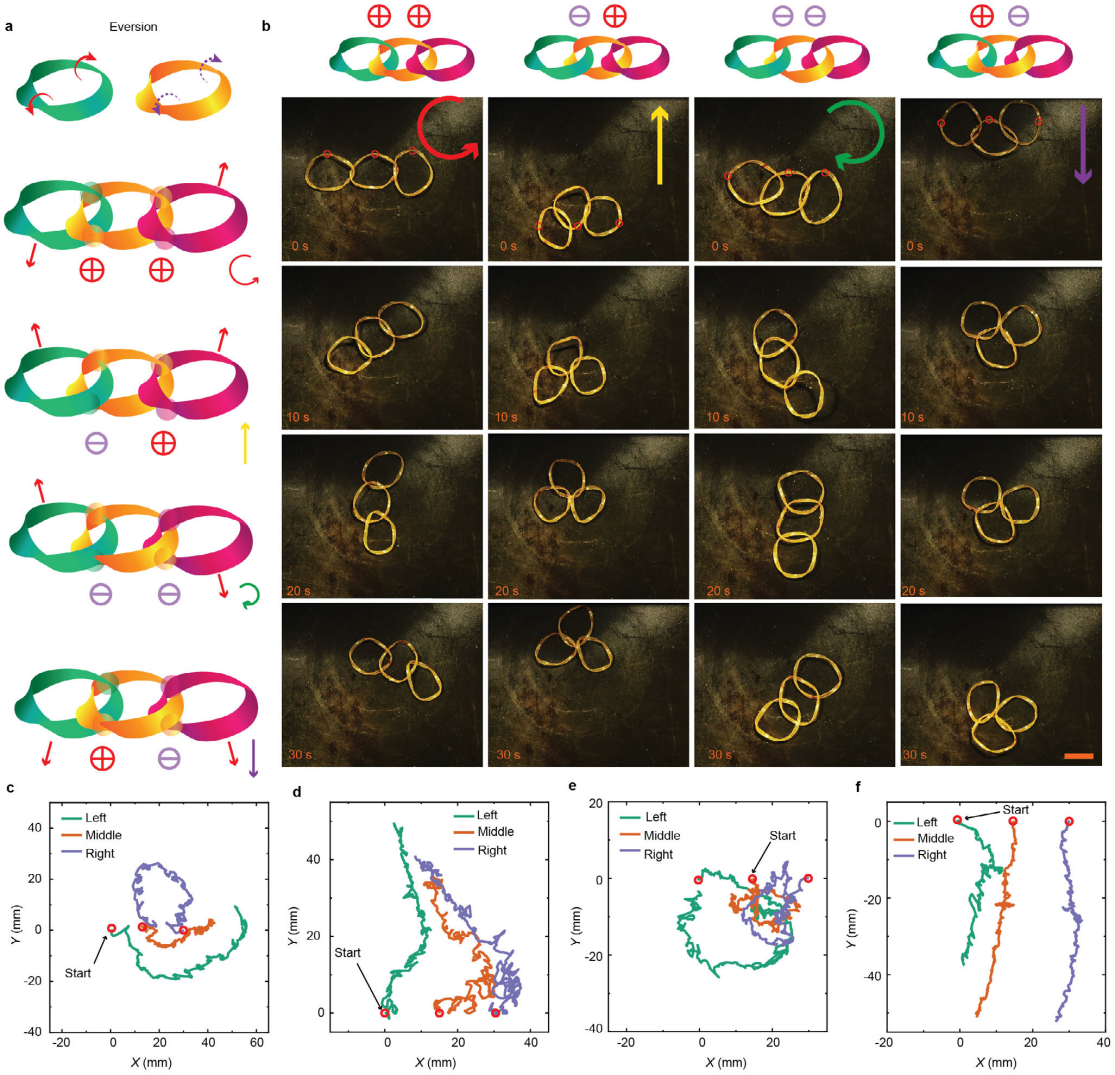
Motion patterns of three connected rings. a) Force analysis in different connections. b) Snapshots of three‐ring clusters with different connections. The red circles in the snapshots highlight the tracking points. Trajectories of three rings based on c) ⨁⨁, d) ⊖⨁, e) ⊖⊖ and f) ⨁⊖ connection modes. The red circles indicate the starting position of the locomotion. All scale bars are 1 cm.

Here, we apply the same connection rule to the linkages between three rings with the same rotation direction (all clockwise or anticlockwise) as shown in **Figure** **4**a. There exist four connection scenarios for ring‐knots with *N* = 3: ⨁⨁, ⨁⊖, ⊖⨁, and ⊖⊖ (Figure 4a,b). Tracking the point on each ring uncovers the occurrence of four different types of group translocation. Such group activities follow the same rule as determined by the connections, regardless of the rotation direction of individual rings. When the three rings are connected in the ⨁⨁ mode, the ring group rotates counterclockwise (Figure 4c; Figure S20a, Supporting Information), while ⊖⊖ producing clockwise rotation (Figure 4e and Figure S20c, Supporting Information). After forming the knot, one portion of the left/right ring has been isolated from the ground. The rest segments of the ring touch the ground, scrolling to induce directional movement tendency. While the middle ring stays still upon zero net friction, the rings on two sides induce a torque for the rotation of the entire cluster with specific handedness. Details of the kinetic analysis shown in Figure 4a. Interestingly, in the ⊖⨁ connection mode, the movement direction is upwards (Figure 4d; Figure S20b, Supporting Information), and the ⨁⊖ mode produces downward locomotion (Figure 4f; Figure S20d, Supporting Information). In these two scenarios, the torques generated by the left and right rings act in opposite directions, canceling each other out in rotation and resulting in the directional translocation of the cluster. Further details on the locomotion are given in Figure S21 and Videos S5 and S6 (Supporting Information).

More intricate locomotive modes can be obtained by mixing the rotation handedness of the connected rings. If the knots are connected by left‐, right‐ and left‐rotating rings, the group velocities follow the same rule as described before (Figure S22 and Video S7, Supporting Information). However, if the knots are connected by right‐, left‐, and left‐rotating rings, all connections (⨁⨁, ⊖⨁, ⨁⊖ and ⊖⊖) lead to the rings piling up and no further translocation takes place (Figure S23 and Video S8, Supporting Information). We also studied the motion of connected rings with different diameters and different connections, as well as three‐ring connection based on Cyclic Catenane knot (Figure S24 and S25, Video S9, Supporting Information).^[^ ^45^ ^]^ No consistent behavioral pattern was observed in these experiments. A closely connected structure combining more rings is also possible. Figure S26 and S27 (Supporting Information) show connections with *N* = 4 and *N* = 5. In all‐⨁ connections, both structures show counterclockwise rotation, while all‐⊖ connections exhibit clockwise rotation (Videos S10 and S11, Supporting Information). Other connections do not show a consistent behavioral pattern for directional movement. We ascribe this to the large number of rings engaged in mechanical interaction that eventually leads to an increased complexity of movement.

## 3 Conclusion

**Figure 5:**
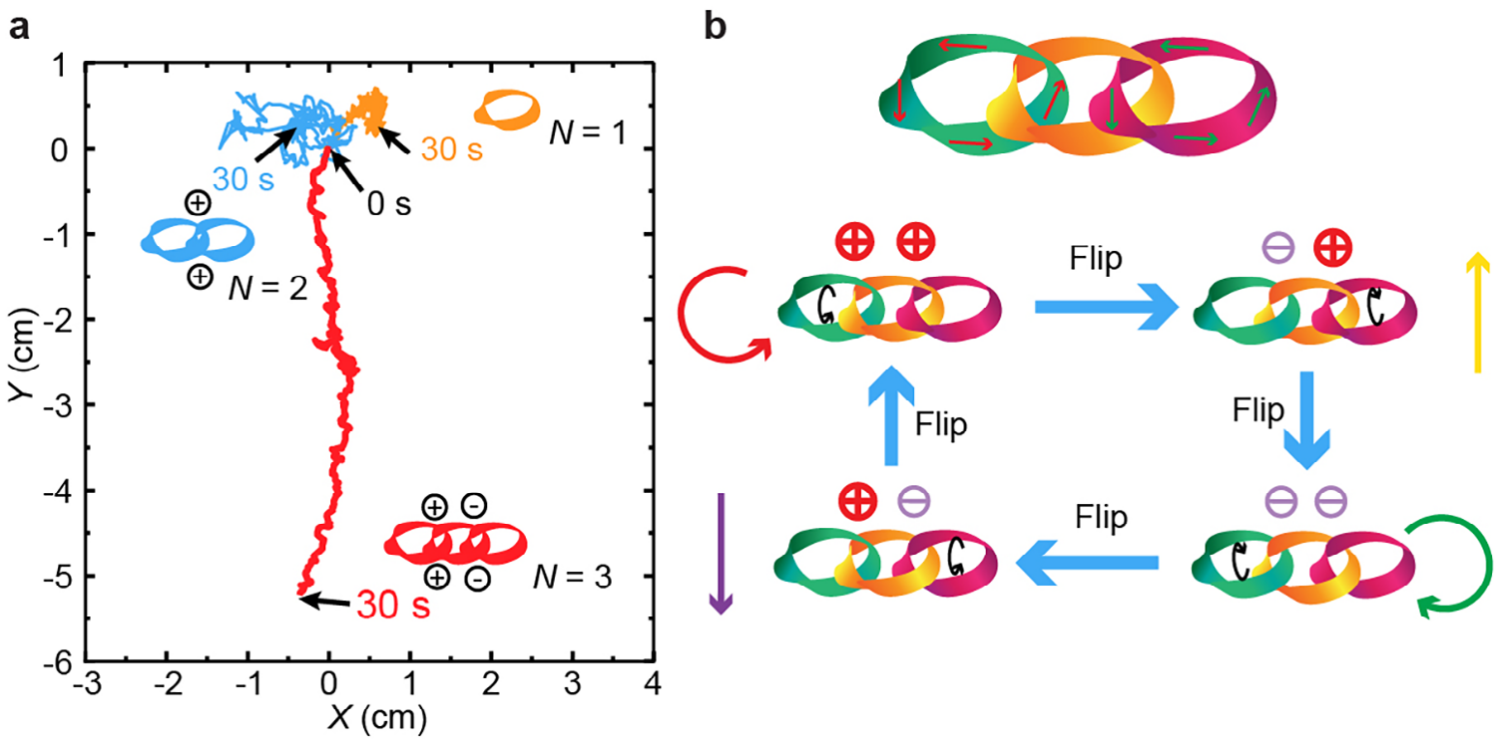
Emergent locomotion of the connected rings. a) The conter‐of‐mass displacement in a single‐ring, two‐ring, and three‐ring knots. The period of position tracking is 30 s and time interval between two data points 33 ms. b) The post‐programmability. Through flipping the rings, one can control the handiness at each connection, yielding different types of locomotion.

ZEEM self‐eversion allows self‐sustained motion of soft‐matter rings under heat gradient,^[^ ^46^ ^]^ operated under a non‐equilibrium, dissipative mechanism. The twisted surfaces provide friction anisotropy at the contacting interface during eversion, thus dictating the handedness of rotation of an individual ring. Hence the basic characteristic of a single ring is the rotation of its circumference without controllable net displacement of the center of mass (**Figure** **5**, orange trajectory). Connecting two rings into a closed loop brings about mechanical interaction between them via two crossing points. This interaction, together with net friction from the ground substrate, creates a mechanical torque imposed onto the ring pair, bringing about the rotation of the entire structure. Each ring is freely rotating either clockwise or anticlockwise, yet the rotation of the ring pair is independent of the rotation direction of the individual rings and is dictated by the mechanical interaction between the rings. However, no controlled net translocation can be obtained in such a two‐ring structure (Figure 5, blue trajectory). In the case of three connected rings, the structure can perform unidirectional net translocation (Figure 5, red trajectory), but only when the three rings are connected with ⊖⨁ or ⨁⊖ patterns (see Figure 3f for definitions). The ⊖⊖ and ⨁⨁ connections result in the rotation of the cluster with defined handedness, inducing no net translocation. In previous reports,^[^ ^5^, ^34^ ^]^ the motion of individual rings was unchangeable or mostly determined by defects in the prepared structures. This study shows that rather than the twisting direction of the individual rings, the handedness of connection between them provides opportunities to program the locomotion of the clustering rings as a group. Importantly, the group exhibits emergent properties that are not achievable without mechanical interactions between its constituents.

The post‐programming approach offers a simple yet effective way to control motion direction by flipping the ring, eliminating the need for disassembly or re‐bonding the structures (Figure 5b). This capability significantly enhances the adaptability and reusability of soft actuators. By reversing the handedness (⨁ or ⊖) through flipping, the system can switch between different locomotion modes, i.e., forward, backward, clockwise, and anticlockwise movements which are listed in Figure 4. For the details of the post‐programming, see Video S12 (Supporting Information).

We have systematically investigated how individual movements influence the collective motion of interacting ring structures. From a bioinspiration point of view, self‐motion far away from thermal dynamic equilibrium is crucial for autonomous robotic functions. Thus, we built our physical model system around ZEEM‐driven rings exhibiting self‐sustained motions. We used liquid crystal elastomer strips that were twisted before connecting their end points to form a closed ring, and the resulting structures self‐rotate on a hot plate due to the heat gradient. Starting with single rings, we examined the effects of the number of twists and the complexity of knot structures on their rotational behavior. We then mechanically connected two rings to study the impact of different connection modes on their movement. While controlled collective rotations can be induced in such two‐ring structures, they show no pre‐programmable net translocation of the center of mass. Expanding our study to three‐ring structures, we found that the center of mass of the entire cluster can undergo a translocation. By controlling the handedness at the crossing points, we can pre‐program the locomotion, i.e., turning left and right, moving forward and backward. Although the physical model is based on ZEEM self‐rotating rings, the locomotive patterns of closely connected rings uncover diverse interactions between soft‐bodied objects through mechanical coupling and a rich level of programmability of the dissipative motions. The results provide insight into self‐sustained motions in responsive soft matter and provide a useful tool to program emergent properties into small‐scale soft robotic constructs.

## Conflict of Interest

The authors declare no conflict of interest.

## Author Contributions

H.G. conceived the idea. H.Z. supervised the project. H.G. performed experiments and analyzed the data. H.G. and H.Z. wrote the manuscript with the help of A.P.; K.L. conducted the modeling. All the authors discussed and contributed to the project.

## Supporting information



Supporting Information

Supplemental Video 1

Supplemental Video 2

Supplemental Video 3

Supplemental Video 4

Supplemental Video 5

Supplemental Video 6

Supplemental Video 7

Supplemental Video 8

Supplemental Video 9

Supplemental Video 10

Supplemental Video 11

Supplemental Video 12

## Data Availability

The data that support the findings of this study are available from the corresponding author upon reasonable request.
