# Supplementary material for: Emergent Locomotion in Self‐Sustained, Mechanically Connected Soft Matter Rings
Source: Adv Mater. 2025 Apr 30;37(26):2503519. doi: 10.1002/adma.202503519 (PMC12232239; doi:10.1002/adma.202503519)
Supplement: Supplementary file 1 — Supporting Information [file ADMA-37-2503519-s012.pdf]

ADVANCED MATERIALS

Supporting Information

for *Adv. Mater.*, DOI 10.1002/adma.202503519

Emergent Locomotion in Self-Sustained, Mechanically Connected Soft Matter Rings

Hongshuang Guo, Kai Li, Arri Priimagi and Hao Zeng**

Supporting information for

Emergent locomotion in self-sustained, mechanically connected soft matter rings

Hongshuang Guo,[1]* Kai Li,[2] Arri Priimagi,[1] Hao Zeng[1]*

[1] Faculty of Engineering and Natural Sciences, Tampere University, P.O. Box 541, FI-33101 Tampere, Finland.

[2] Department of Civil Engineering, Anhui Jianzhu University, Hefei 230601, China.

Correspondence to: hongshuang.guo@tuni.fi, hao.zeng@tuni.fi.

This PDF file includes:

1. Materials and Methods
2. Figures S1 to S27
3. Supplementary methods for modeling
4. References
5. Captions for Supplementary Videos S1-S12

Additional supplementary material for this manuscript includes:

Videos S1-S12

1. Materials and Methods

LCE Material preparation in brief. 1,4-Bis-[4-(6-acryloyloxyhexyloxy)benzoyloxy]-2-methylbenzene (99%, RM82) was purchased from SYNTHON Chemicals GmbH & Co. 1-amino-3-(dimethylamino)propane and cystamine dihydrochloride were purchased from TCI. Cystamine was obtained by treating the cystamine dihydrochloride with NaOH, and extracted with dichloromethane. 1,4-Diiodotetrafluorobenzene was purchased from Fluorochem Ltd. All reagents and chemicals were used as received without further purification.

Film preparation. Cells were prepared by gluing two coated polyvinyl alcohol (PVA, 5% water solution, 4000 RPM for 1 min and baked at 100 ºC for 10 min) glass substrates together with 550 µm microspheres (Thermo scientific) that acted as spacers to determine the LCE film thickness. Liquid crystal mixtures were prepared by mixing 1 mmol RM82, 0.3 mmol cystamine, 0.4 mmol 1-amino-3-(dimethylamino)propane, and 0.2 mmol 1,4-Diiodotetrafluorobenzene at 100 ºC until all components melted into a clear liquid. The mixture was infiltrated into the cells via capillary effect at 90 ºC and cooled down to 60 ºC. The cells were put in an oven to allow the Aza–Michael addition reaction for oligomerization for 4 h at 60 ºC and then heated to 90 ºC to allow the reaction to proceed for another 20 h. The polymerized cells were soaked in water to dissolve the PVA, then opened using a blade underwater. The sample was dried in the air and strips with the desired dimensions were cut from the film.

Monodomain sample preparation. To align the initially disordered LCEs, the samples were heated to 60 °C, uniaxially stretched (typically by 100 %), and subsequently cooled to room temperature while stretching. The aligned and shape-programmed LCEs were kept at room temperature for at least 72 h before characterization and testing. For obtaining the ring structures, two ends of a twisted strip were glued together using Loctite Precision glue. The volume of glue should not exceed *ca.* 10 micro litter to avoid the generation of defect that influence the self-sustained eversion and reproducibility of the sample.

Actuation measurements. Thermal actuation measurements were performed by placing aligned

samples on a black anodized aluminum sheet on a hotplate. The samples were heated from 30 to 110 °C by increasing the temperature at 10 °C intervals while imaging them with a microscope.

Material characterization. DSC measurements were performed with a NETZSCH DSC 214 polyma instrument at a heating/cooling rate of 10 °C min^{-1}. The measurements were performed using 7–12 mg sample weight under 1 bar nitrogen atmosphere (flow rate of 20 mL min^{-1}) at the temperature range between -50 – 150 °C. Tensile tests were performed on the Instron 5567 universal tensile tester using a 100 N load cell. The dimension of the rectangular film was measured with a digital caliper. The tensile speed was set at 5 mm min^{-1}. Each measurement was repeated three times. The alignment of the monodomain sample was characterized by a polarized optical microscope (Zeiss Axio Imager) by imaging the samples at 0 and 45° angles between the polarizer/analyzer axes. Wide-angle X-ray scattering (WAXS) was conducted using a Xenocs Xeuss 3.0 SAXS/WAXS system (Xenocs SAS, Grenoble, France) equipped with an area detector (Eiger2 R 1M, Dectris AG, Switzerland). The system includes two microfocus X-ray sources with sealed tubes targeting either Cu or Mo. The beams, collimated by a multilayer mirror, produce parallel beams with nominal wavelengths of 1.542 Å (combined Cu $K\text{-}_{\alpha 1}$ and Cu $K\text{-}_{\alpha 2}$ characteristic radiation) for room-temperature measurements, and 0.711 Å (combined Mo $K\text{-}_{\alpha 1}$ and Mo $K\text{-}_{\alpha 2}$ characteristic radiation) for temperature-controlled measurements. The sources operate at 50 kV with currents of 1.0 mA (Mo target) or 0.6 mA (Cu target). Measurements were conducted in a vacuum chamber, with the beam size at the sample position set to 0.7 mm × 0.7 mm. Calibration of the sample-to-detector distance was performed using a LaB_6 standard sample. For WAXS, the sample-to-detector distances were 55 mm for room temperature and 150 mm for temperature-controlled conditions. WAXS data were collected over a temperature range of 25–100 °C, and background subtraction was performed using data from an empty chamber. Azimuthal profiles were obtained by integrating over a q

range of 1.3 $Å^{-1}$ to 1.5 $Å^{-1}$ and were fitted with a Gaussian function.

Imaging. All photographs and supplementary videos were taken with a Canon 5D Mark III camera equipped with a 100 mm lens. Surface temperature changes were recorded with an infrared camera (FLIR T420BX) equipped with a close-up (2×) lens. Kinovea program was used to monitor the motion of the trajectory of the vertex, yielding distance and time data at 33 ms time intervals as coordinates for the x- and y-axis.

2. Supplementary Figures

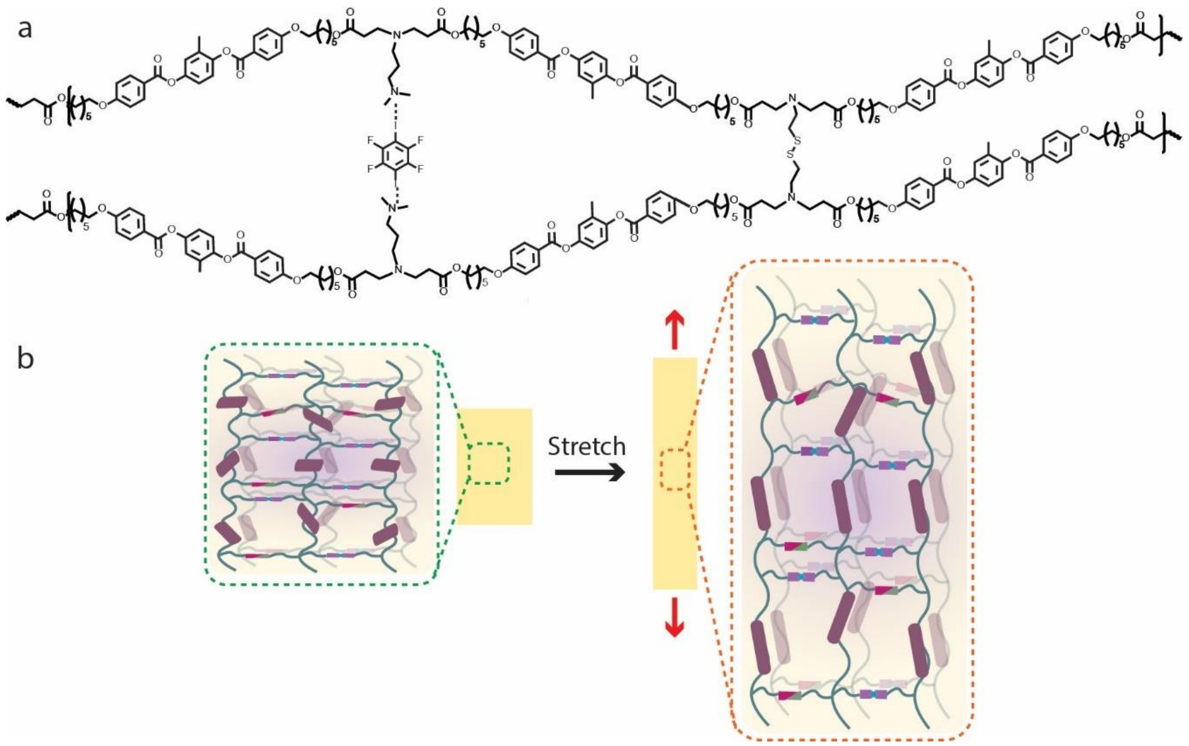

Figure S1. Preparation of the LCE soft actuator. (a) The depicted supramolecular network. (b) Schematic illustration of the stretching-induced orientation of the LCE. The as-prepared film is in isotropic, polydomain state, and uniaxially aligned upon stretching. The sample kept in the stretched state at room temperature for three days to achieve reversible actuation.

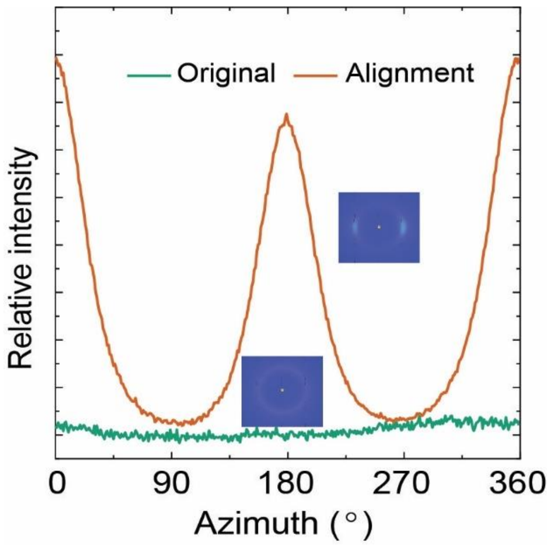

Figure S2. WAXS data. 1D azimuthal scan profiles of the LCE strip before and after stretching (100 %). Insets: Corresponding 2D WAXS patterns.

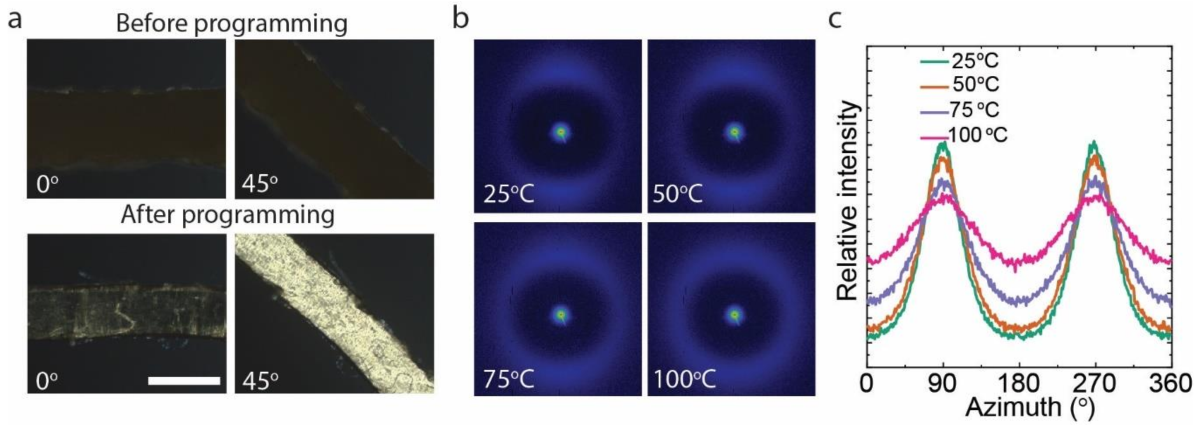

Figure S3. Liquid crystalline alignment. (a) Polarized optical micrographs of the LCE film before programming and after programming. The sample directors are placed at 0° and 45° angles in respect to the polarizer/analyzer. Scale bar is 1 cm. (b) WAXS patterns of the aligned LCE film after 100% stretching during heating at different temperature and (c) their corresponding azimuthal profiles.

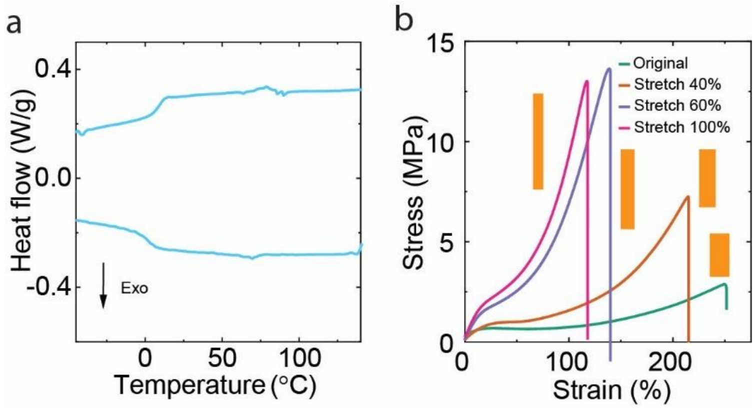

Figure S4. Materials thermodynamic and mechanical properties. (a) DSC curves of the LCEs before stretching. The DSC data is taken from the second cooling cycle at a heating/cooling rate of 10 °C/min. (b) Stress-strain curves of the LCE strip after stretching to different lengths.

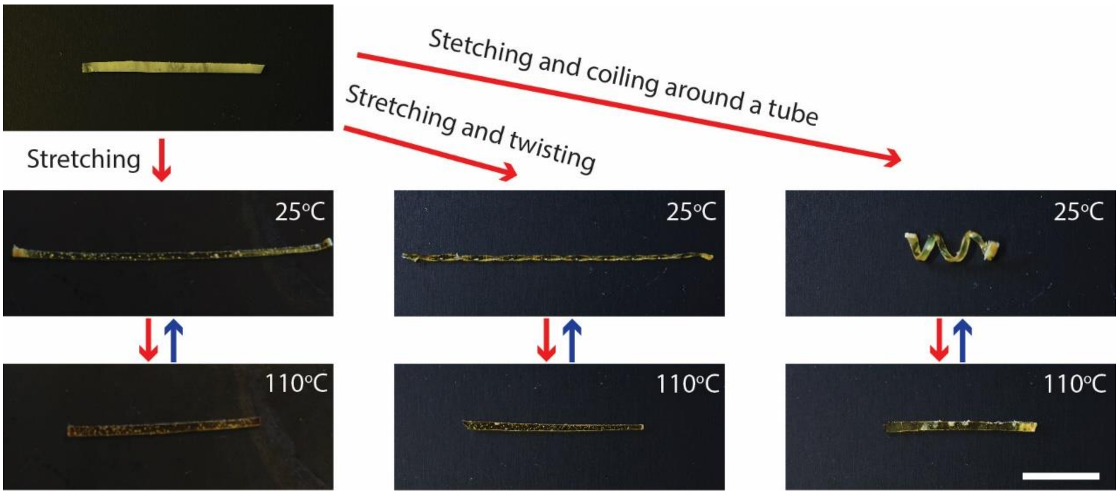

Figure S5. Programming the shape changes. Photographs of shape-programmed LCE strips. The shape programming, *i.e.*, stretching, stretching and twisting, stretching and coiling around a tube, are done at 60 °C. After the shape-programming, the LCE can reversibly deform upon heating and cooling. Scale bar: 1 cm.

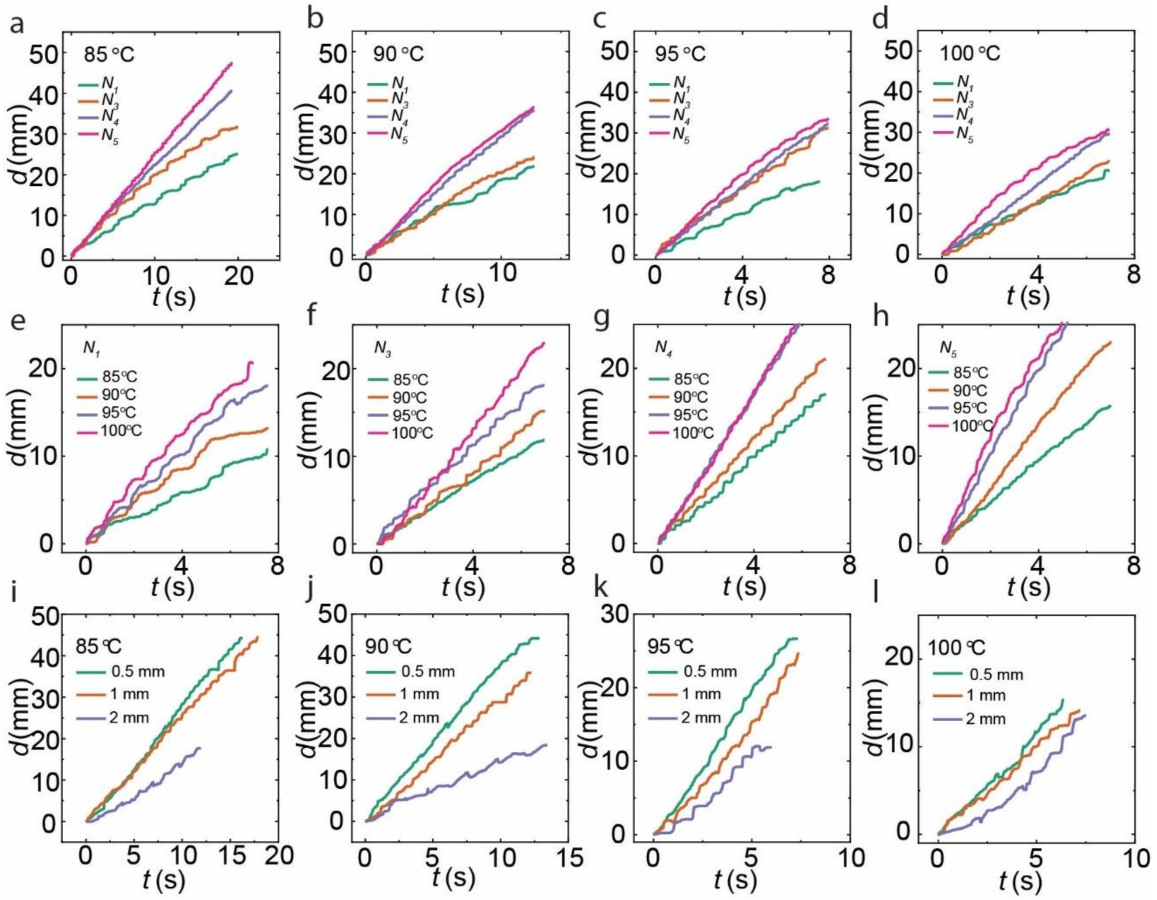

Figure S6. Self-sustained motion of twisted strips. (a-d) Rolling distance over time of different twisted strips on a hot plate at different temperatures. (e-h) Rolling distance over time of the twisted LCE strips at various temperatures. (i-l) Rolling distance over time of the LCE strips with different width on at different temperatures.

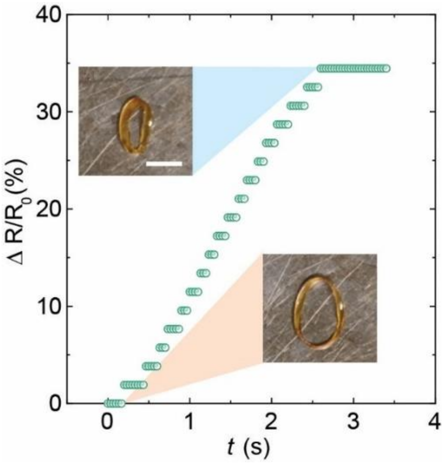

Figure S7. Kinetics of heat-induced shrinkage. Radius change upon heating, when the LCE ring is set on a 120 °C hot plate. *R*, the radius of the deformed ring. R_0, the original radius. Insets: photographs of the static strip ring before (bottom) and after (top) shrinking. Scale bar: 1 cm.

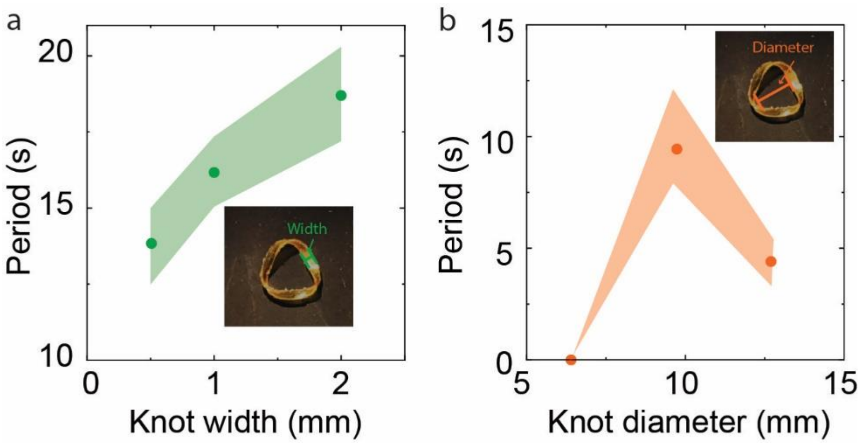

Figure S8. Eversion speed for different LCE ring dimensions. (a) Eversion period of rings with different widths at 105°C. (b) Eversion period of rings with different diameters at 105°C. The inset depicts the width and diameter of the rings. We define the period of eversion as the time required to complete a 360° eversion from the top view. Error bars indicate standard deviation for n = 3 measurements. The same sample was measured repeatedly.

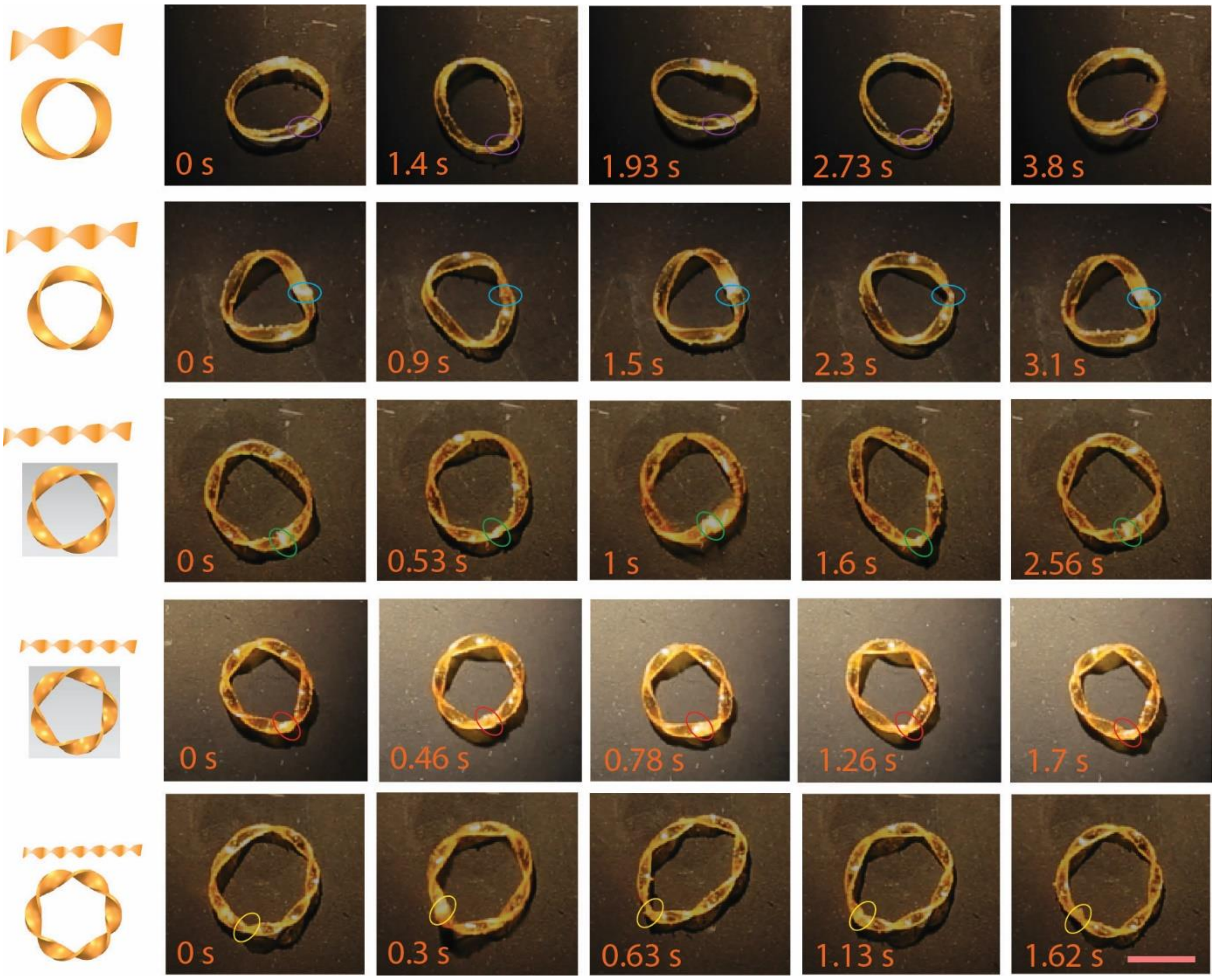

Figure S9. The rotation of a single ring knot. Snapshots of strip rings with different twist numbers. Scale bar: 1 cm.

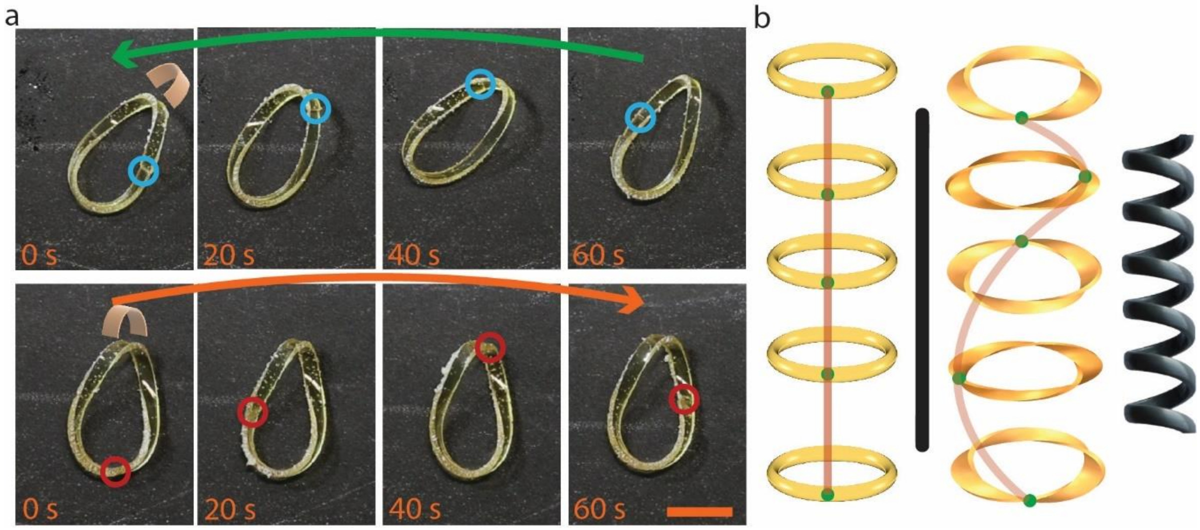

Figure S10. Rotation direction of a single ring. (a) Snapshots of strip rings with opposite twists showing different rotation directions. Scale bar: 1 cm. (b) Schematic diagram illustrating the rotation kinetics of a torus made of a cylinder, versus, a ring made of a twisted strip.

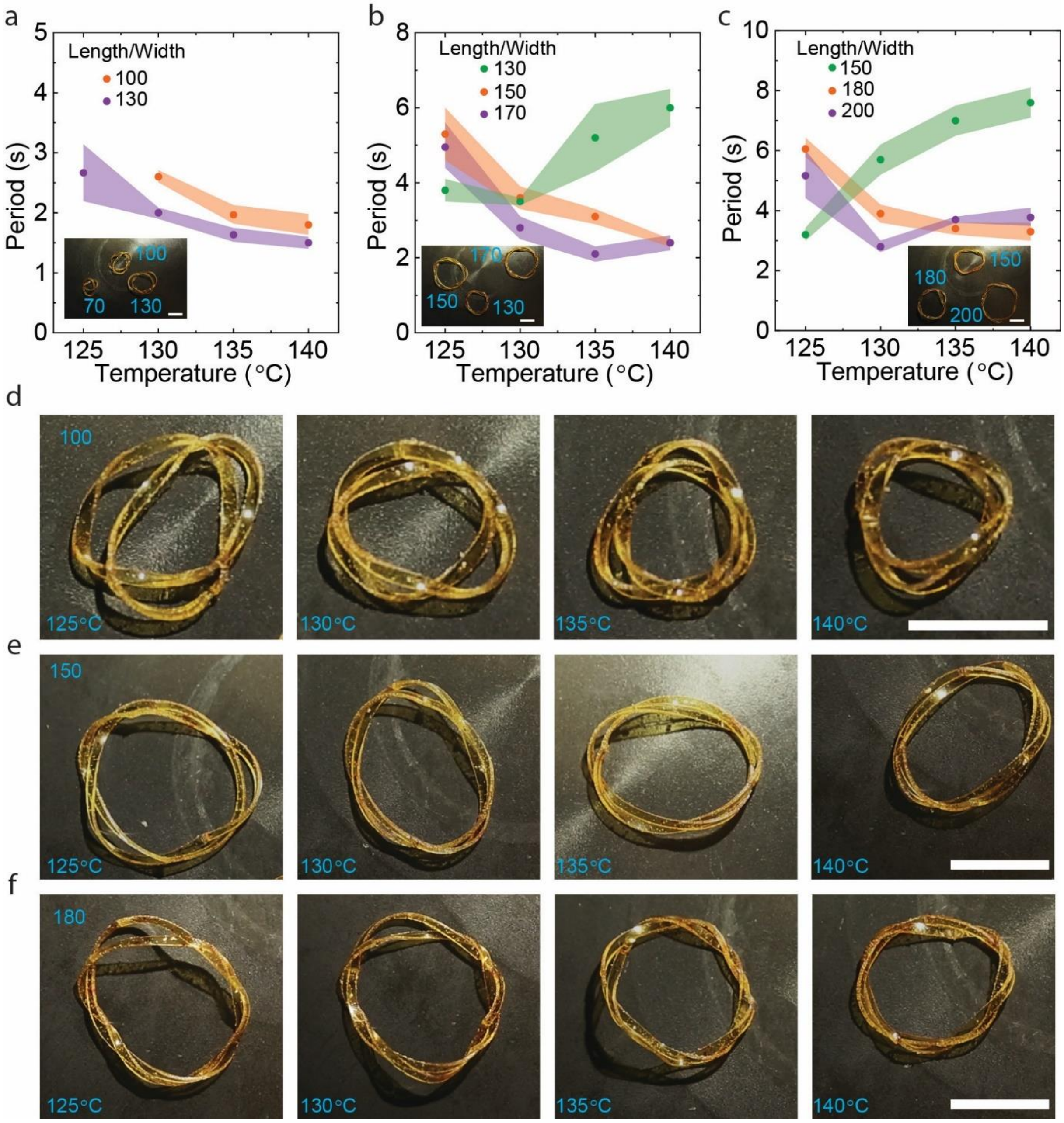

Figure S11. The influence of sample aspect ratio on the eversion. The eversion period of (a) Trefoil knot/3, (b) Pentafoil knot/5, and (c) Septoil knot/7 upon different heating temperature. Note that the Trefoil Knot/3 with an aspect ratio of 70 does not rotate consistently and Trefoil Knot/3 with an aspect ratio of 100 does not rotate at 125°C, no data was obtained. Snapshots of (d) Trefoil knot/3, (e) Pentafoil knot/5, and Septoil knot/7 at different temperature indicating the dimensional change. Error bars indicate standard deviation for n = 3 measurements. The same sample was measured repeatedly. The same sample was measured repeatedly. All scale bars are 1 cm.

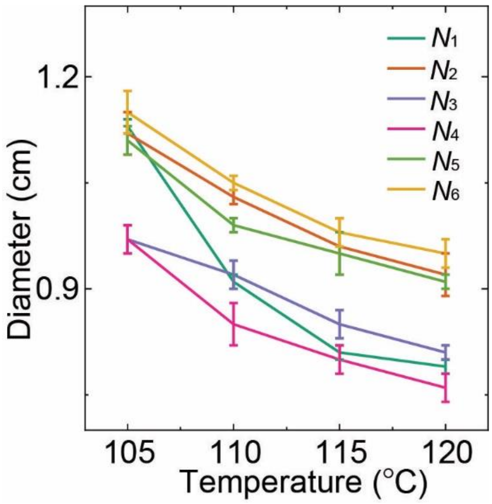

Figure S12. Heat-induced shrinkage. Diameter change of rings with different twist numbers as a function of temperature. Error bars indicate standard deviation for n = 3 measurements. The same sample was measured repeatedly.

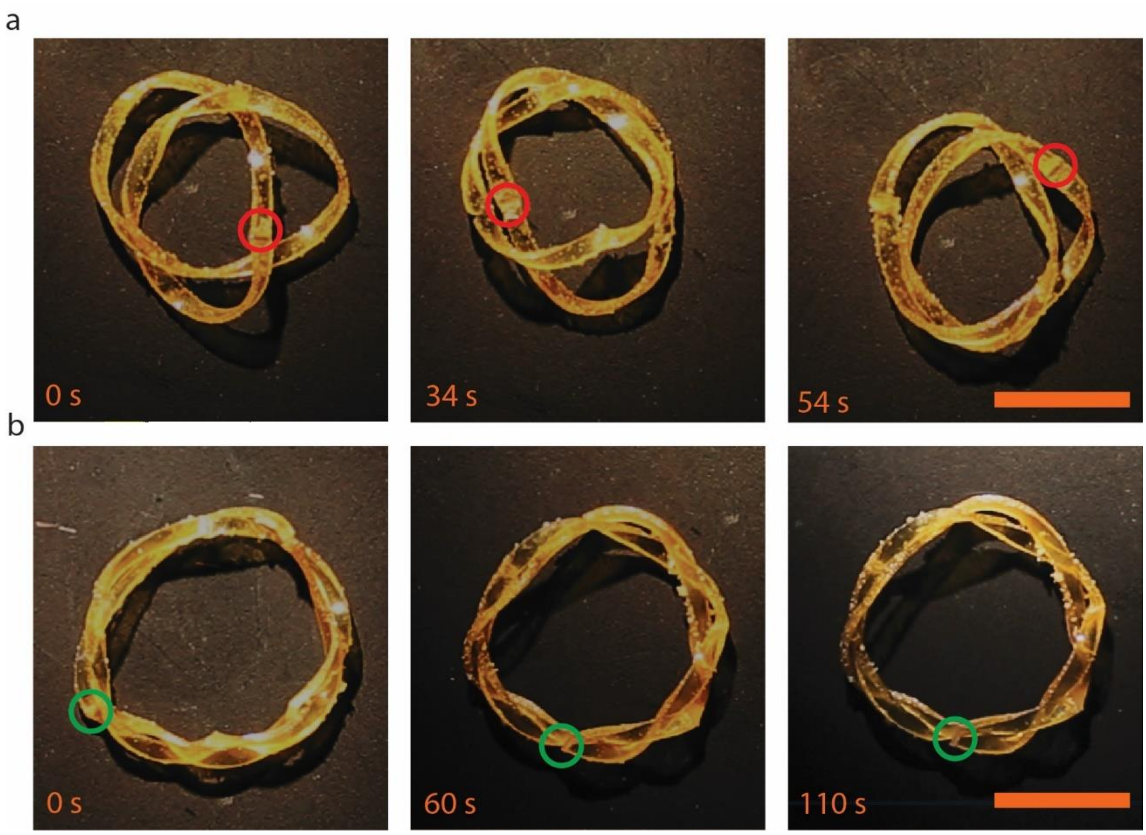

Figure S13. Autonomous rotation of complex knot rings. (a) Snapshots of a Trefoil Knot/3 ring. (b) Snapshots of a Septoil Knot/7 ring. Colored circles indicate the tracking of position during the rotation. All scale bars are 1 cm.

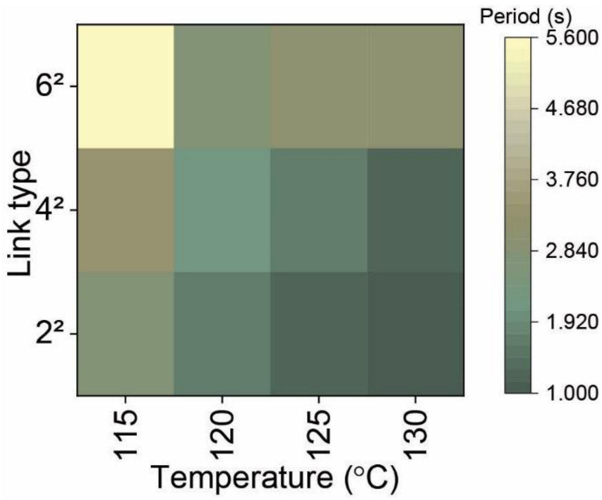

Figure S14. Color map summarizing the eversion period as a function of temperature and the link types.

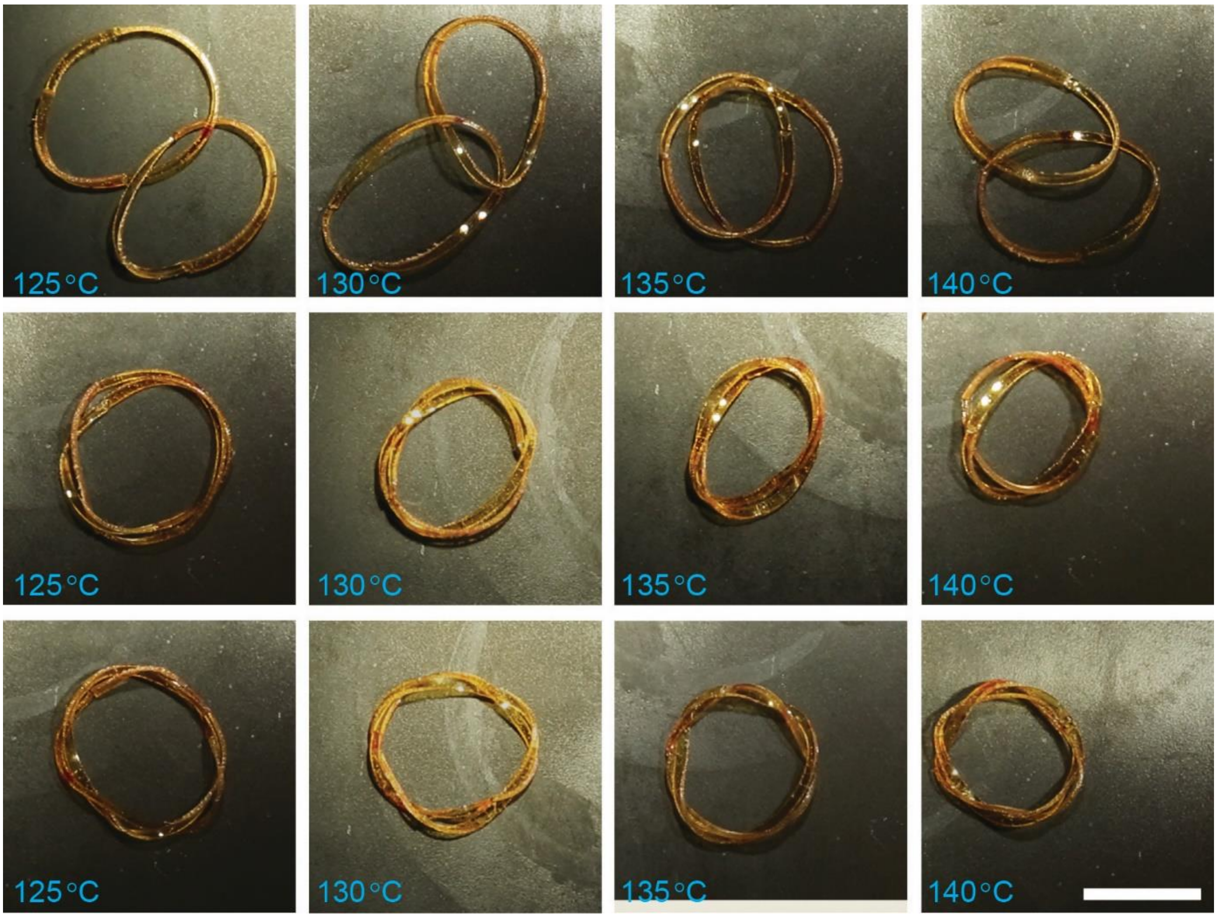

Figure S15. Diameter changes upon elevated temperatures. Snapshots of Hopf Link/2, Solomon Link/4, and Star of David/6 at different heating temperatures Scale bars: 1 cm.

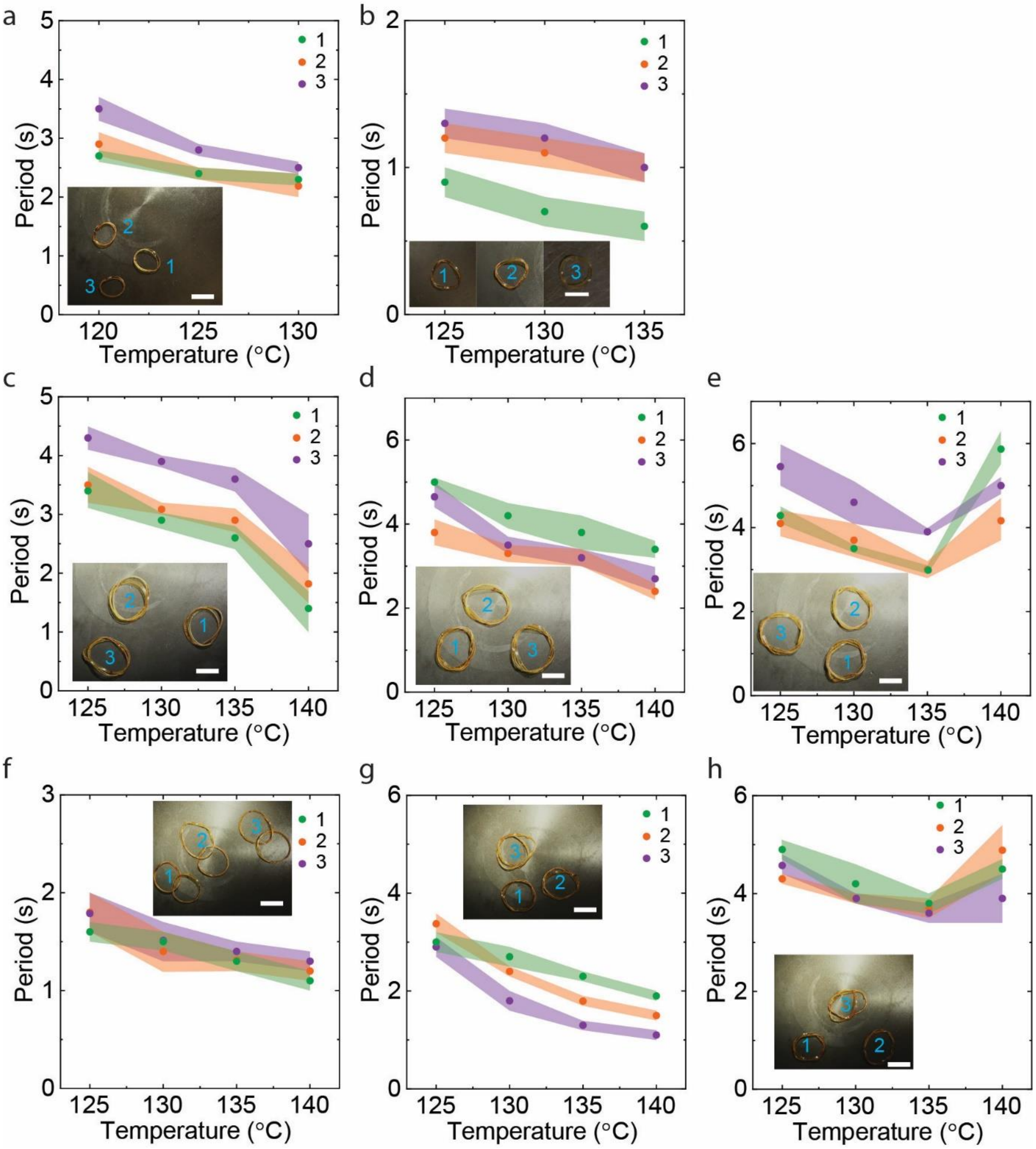

Figure S16. Eversion behaviours across different samples. The eversion period of (a) N_1 ring (aspect ratio: 30), (b) N_3 ring (aspect ratio: 34), (c) Trefoil Knot/3 (aspect ratio: 120), (d) Pentafoil Knot/5 (aspect ratio: 150), (e) Septoil Knot/7 (aspect ratio: 190), (f) Hopf Link/2 (aspect ratio: 50), (g) Solomon Link/4 (aspect ratio: 50) and (h) Star of David/6 knots (aspect ratio: 50) upon different heating temperature. Each data panel records the performances from three individual samples with the same dimension. Insets are photographs of the individual samples. Error bars indicate standard deviation for n = 3 measurements. The same sample was measured repeatedly. All scale bare are 1 cm.

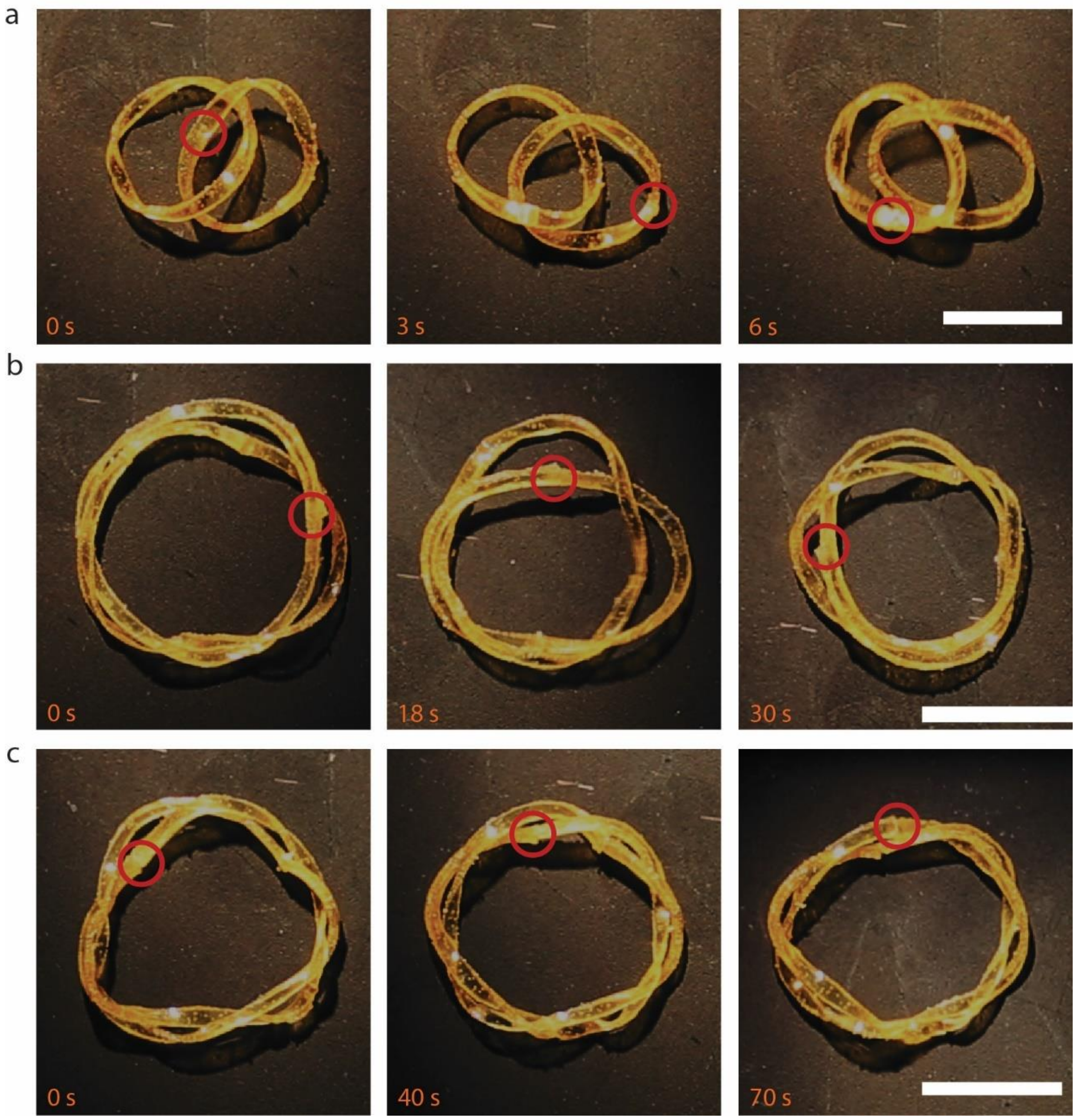

Figure S17. Motion of two connected rings. Snapshots of (a) Hopf Link/2, (b) Solomon Link/4, (c) Star of David/6. Circles indicate the position tracking during the motion. All scale bars are 1 cm.

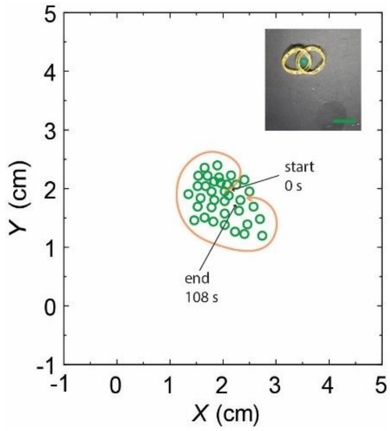

Figure S18. Translocation property. The trajectories of the center of mass in Hopf Link/2 ring. Inset figure depicts the center of the mass as the tracked spot.

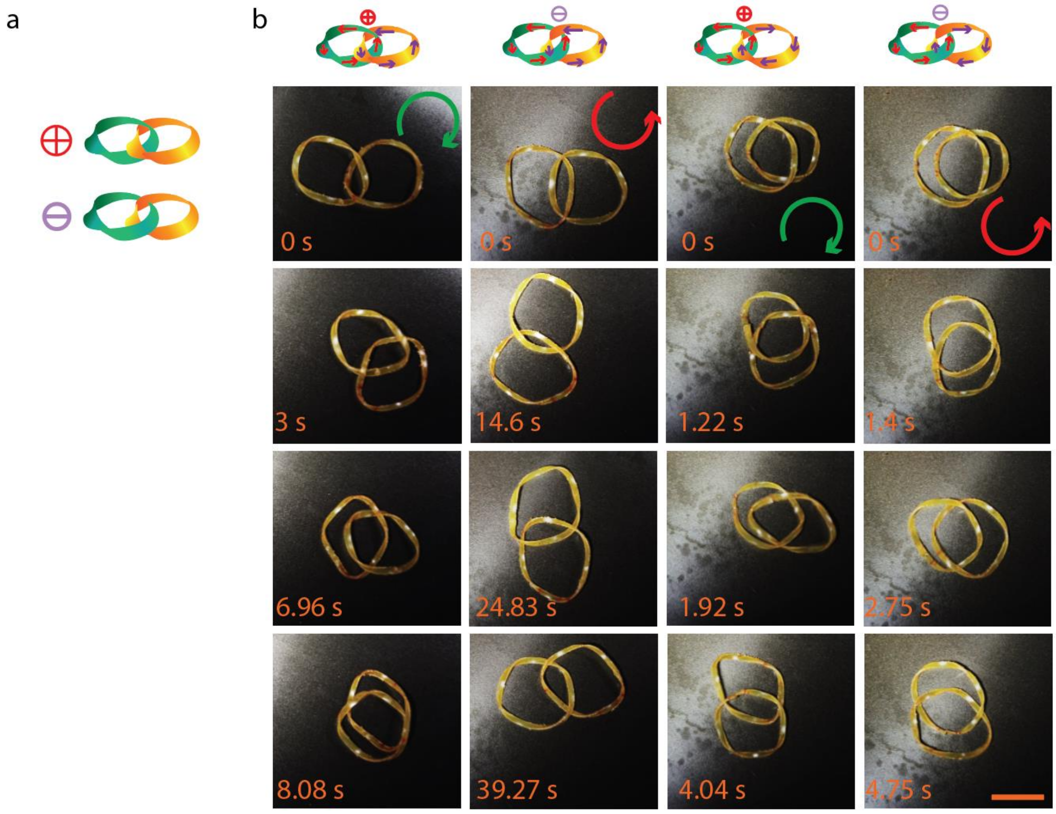

Figure S19. Motion behavior of two connected rings. (a) The definition of the ⊕ and ⊖ connections. (b) Snapshots of the Hopf Link/2 rings, with the red and the green arrow indicating the rotation direction with ⊕ and ⊖ connections and different handedness. All scale bars are 1 cm.

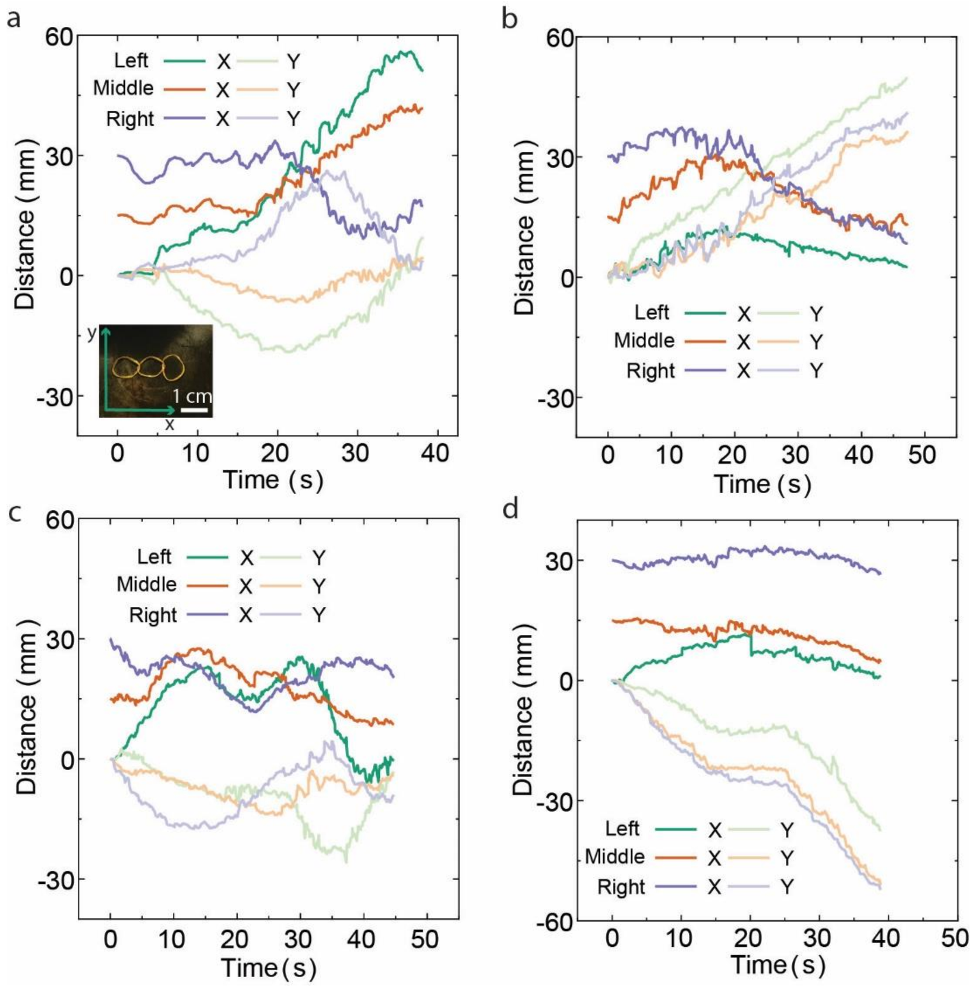

Figure S20. Motion behavior of two connected rings. Tracking of the position of three rings at different time based on different connection modes: (a) ⊕⊕, (b) ⊖⊕, (c) ⊖⊖ and (d) ⊕⊖. The inset in (a) define X and Y direction.

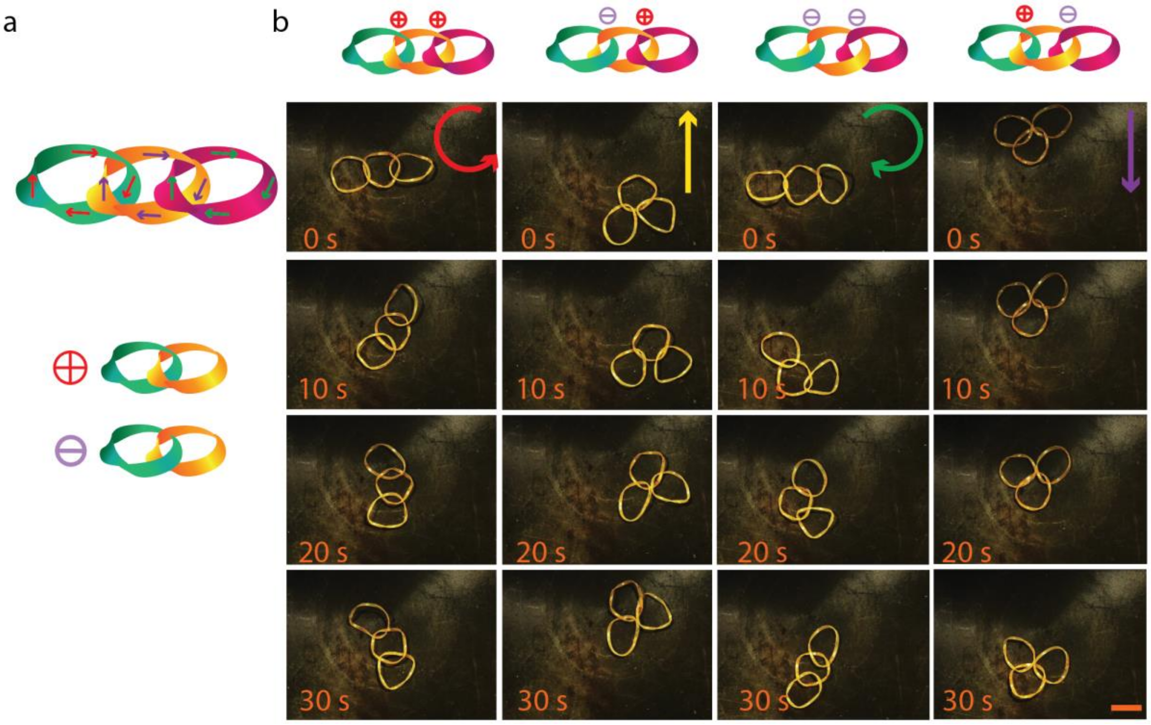

Figure S21. Three connected rings with the same rotation direction. (a) The definition of the connection. (b) Snapshots of ring knots with different connections. All rings rotate clockwisely. The rings are connected with ⊕⊕, ⊖⊕, ⊖⊖and ⊕⊖ modes. Colored arrows indicate the rotation or moving direction of the cluster. All scale bars are 1 cm.

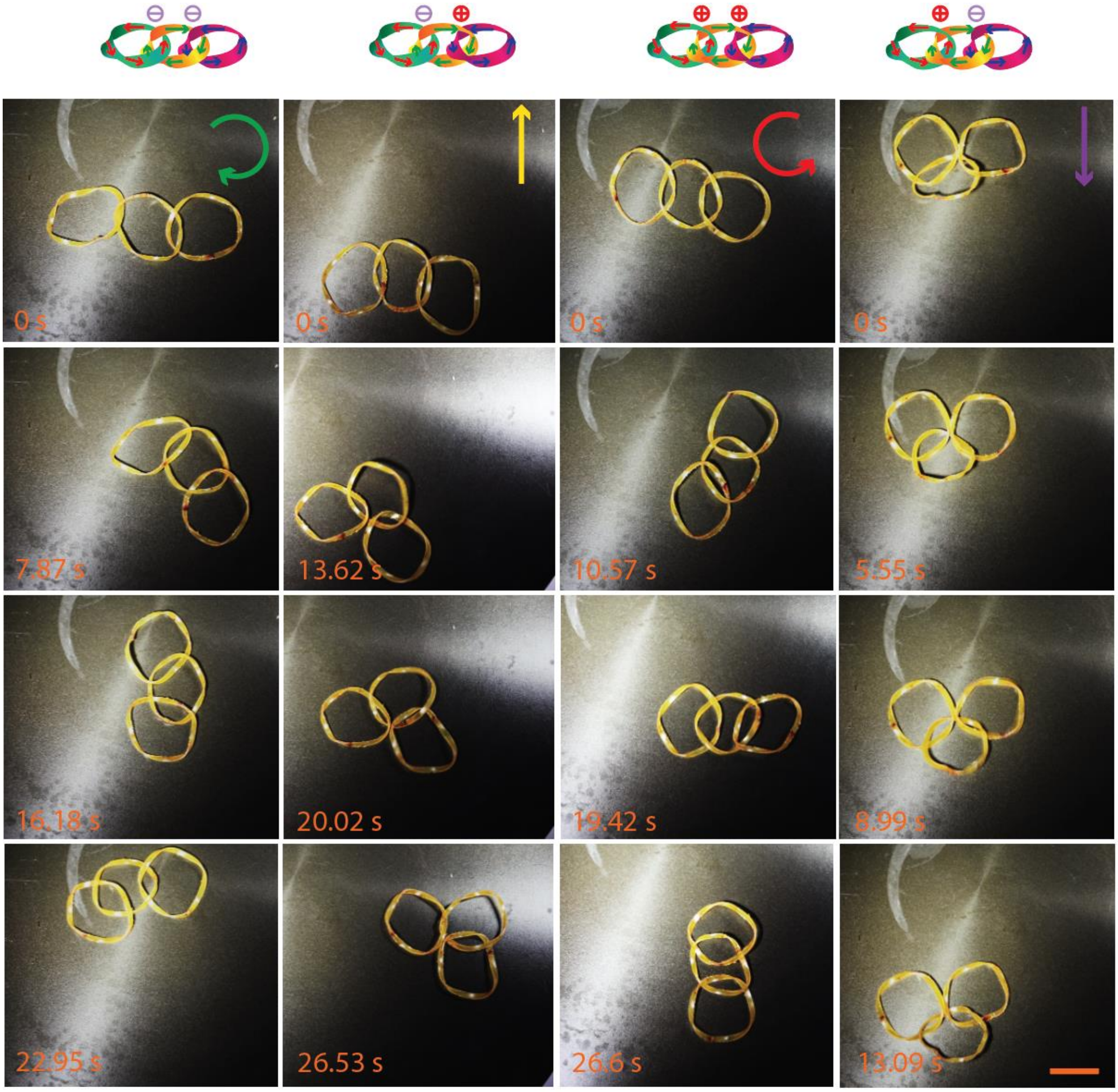

Figure S22. Three connected rings with left-right-left handed rotation. Snapshots of ring clusters with ⊖⊖, ⊖⊕, ⊕⊕ and ⊕⊖ connection modes. Colored arrows indicate the rotation or moving direction of the cluster. Here the rotation directions of the three rings are anticlockwise, clockwise, and anticlockwise, respectively. All scale bars are 1 cm.

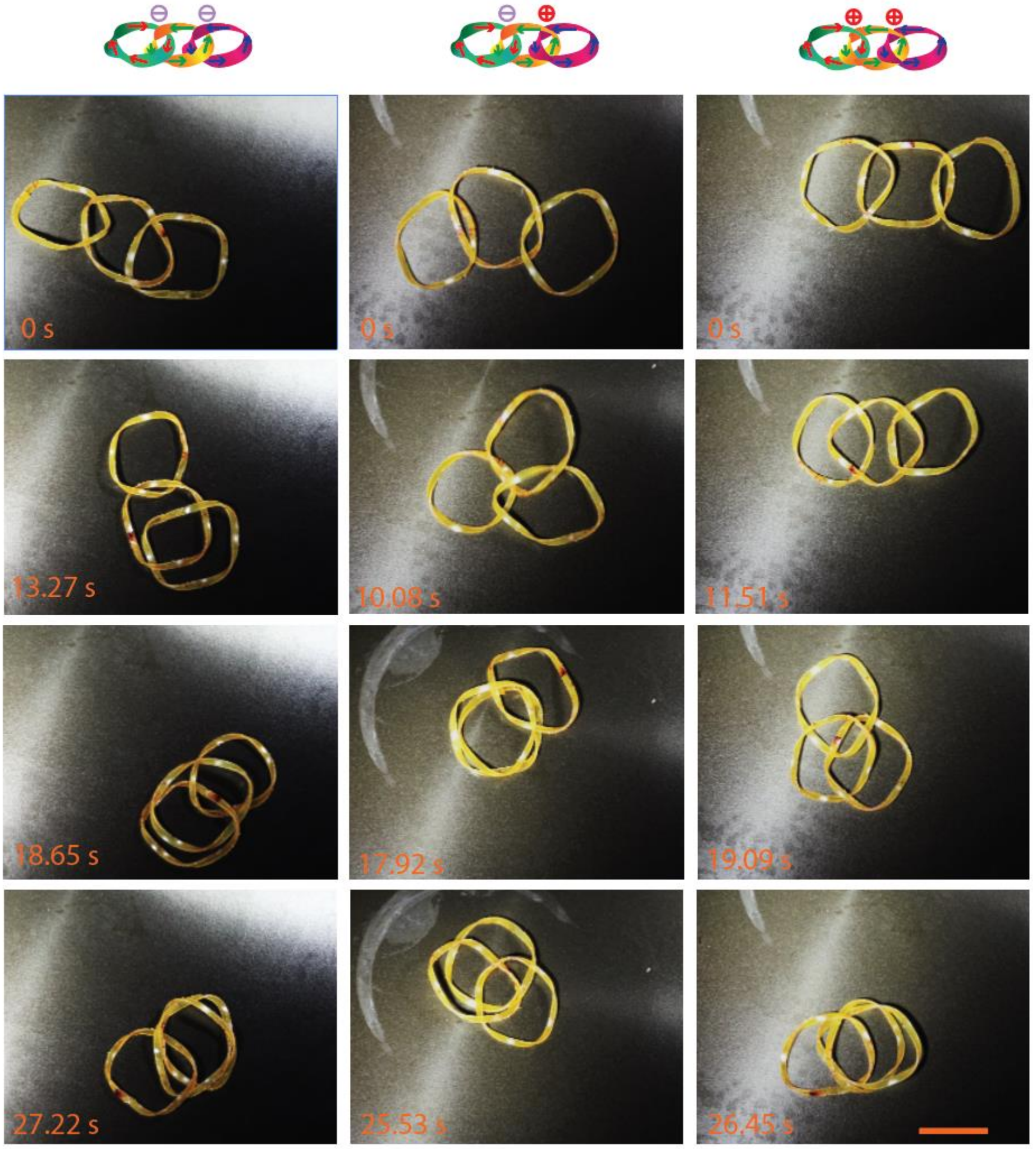

Figure S23. Three connected rings with right-left-left handed rotation. Snapshots of ring clusters with ⊖⊖, ⊖⊕ and ⊕⊕ connection modes. Here the rotation directions of the three rings are clockwise, anticlockwise, and anticlockwise, respectively. All scale bars are 1 cm.

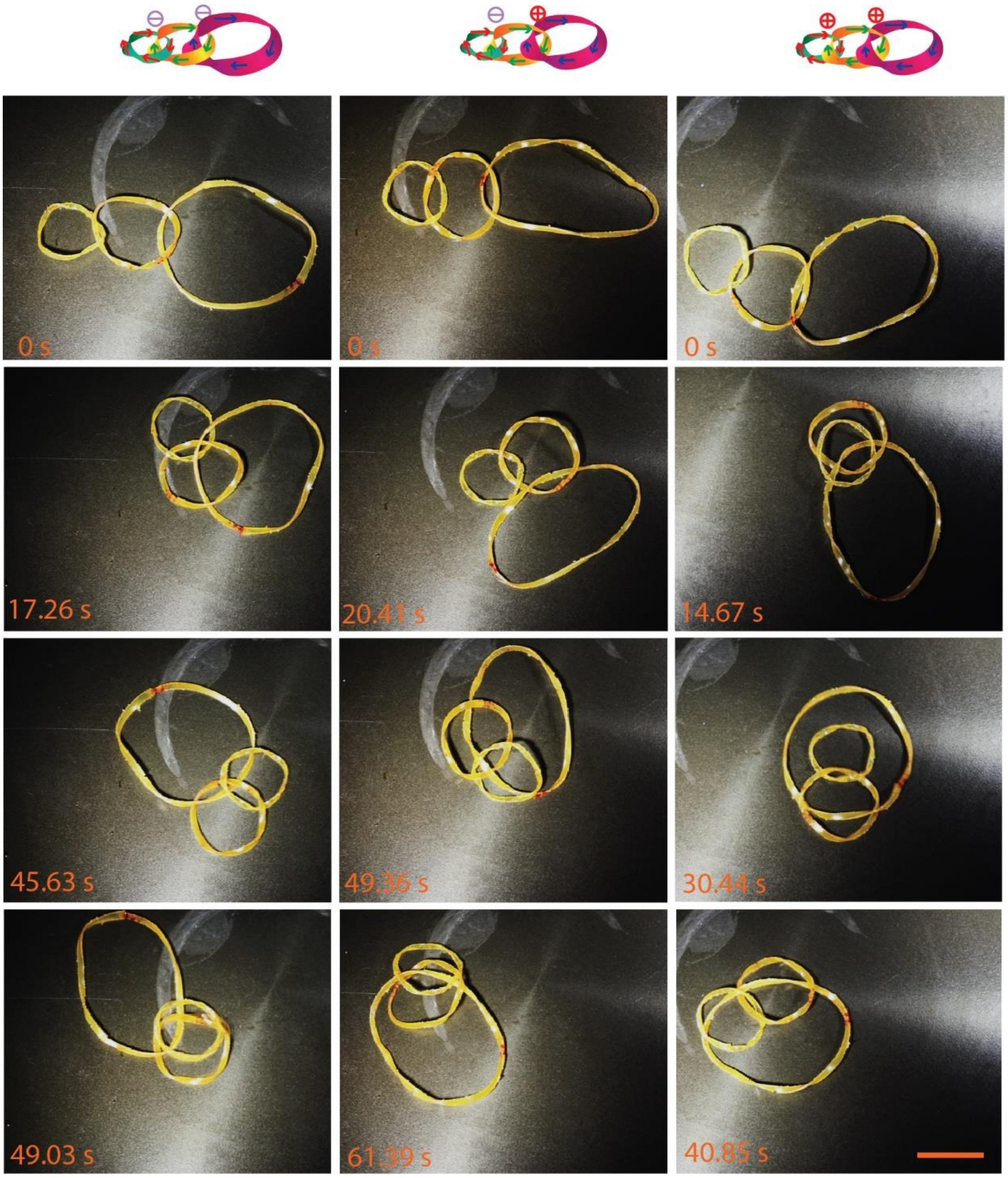

Figure S24. Three connected ring knot with different ring size. Snapshots of ring clusters with ⊖⊖, ⊖⊕ and ⊕⊕ connection modes. Here all three rings rotate clockwise. All scale bars are 1 cm.

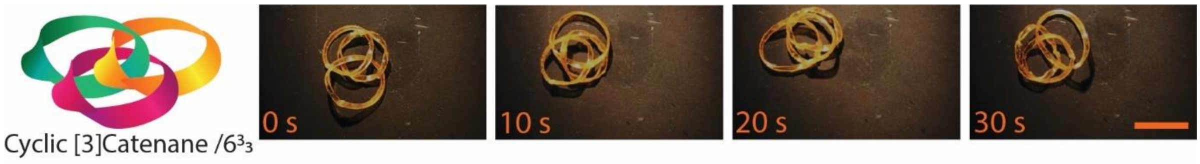

Figure S25. Three linked rings with Cyclic [3]Catenane /6^3_3. Snapshots of the ring cluster. All rings rotate anticlockwisely. All scale bars are 1 cm.

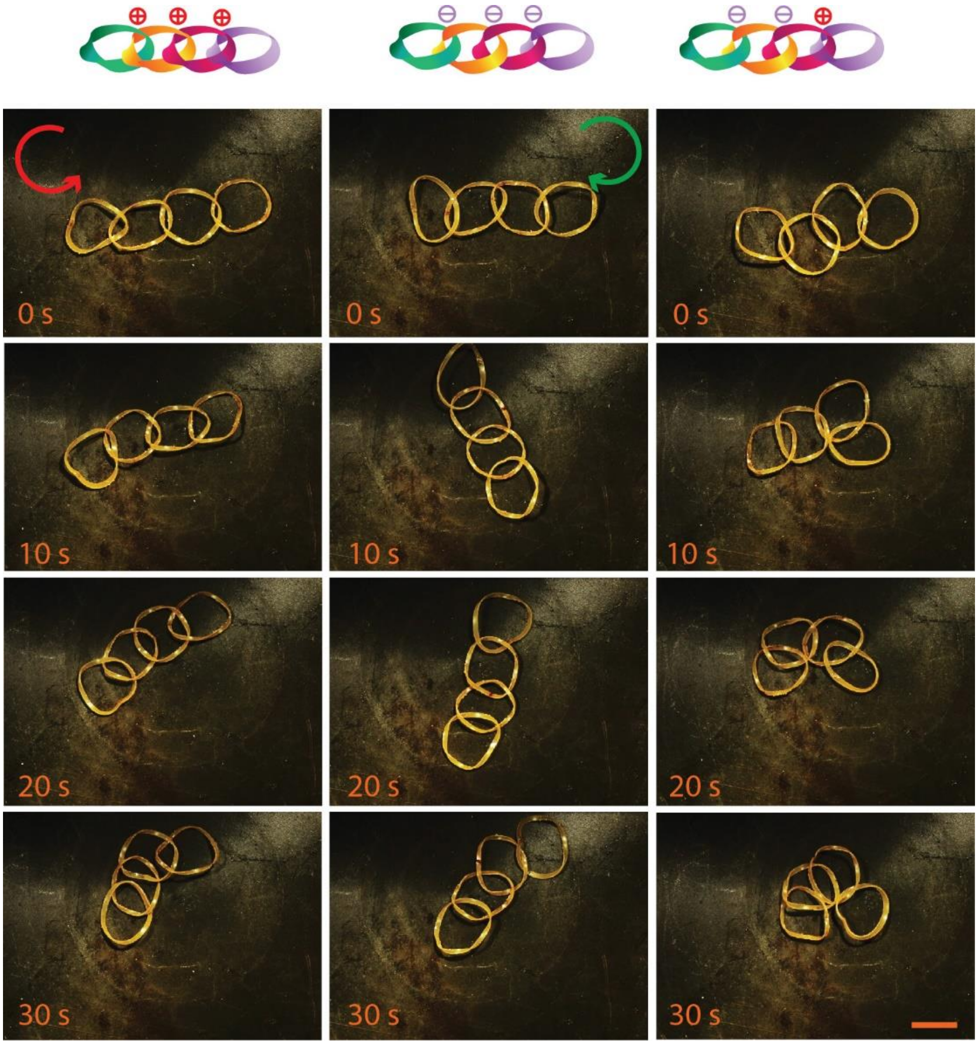

Figure S26. Knot with four connected rings. Snapshots of ring clusters with ⊕⊕⊕, ⊖⊖⊖ and ⊖⊖⊕ connection modes. All rings rotate anticlockwise. The colored arrows indicate the rotation direction of the cluster. All scale bars are 1 cm.

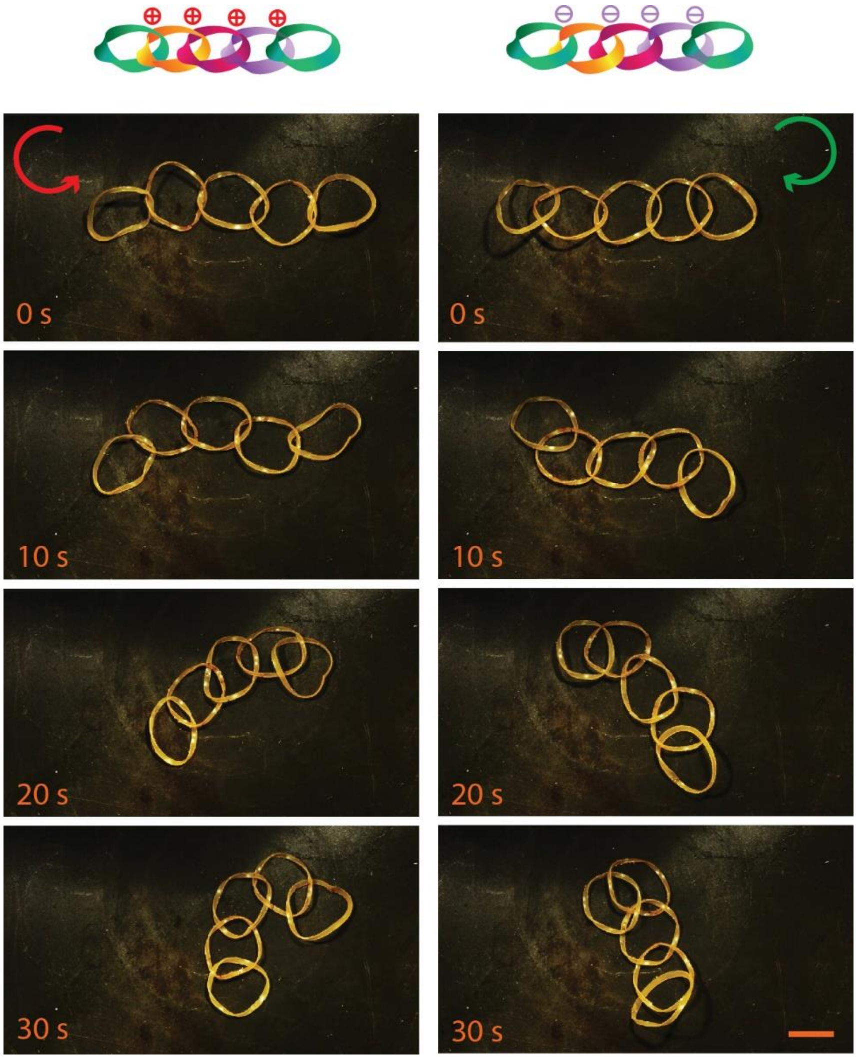

Figure S27. Knot with five connected rings. Snapshots of ring clusters with ⊕⊕⊕⊕ and ⊖⊖⊖⊖ connection modes, respectively. All rings rotate anticlockwise. The colored arrows indicate the rotation direction of the cluster. All scale bars are 1 cm.

3. Supplementary methods for modeling.

3.1 Initial stage of a twisted torus

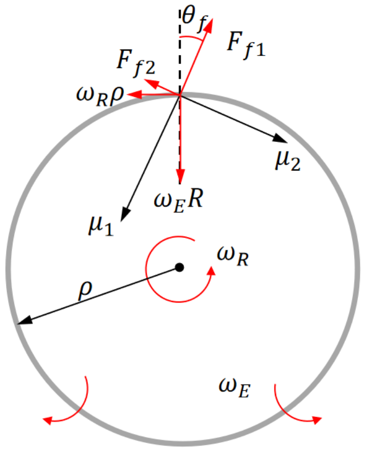

Figure S24. Heat-driven eversion and rotation of a twisted torus with helical threads on its surface on a hot plate. the radius of the cross section is R, the curvature radius of the tours is ρ. The included angle between helical thread and radial direction is θ_f, The anisotropic friction coefficients of threads are μ_1 and μ_2, respectively. For the steady movement, the angular velocity of eversion is ω_E, and the angular velocity of rotation is ω_R.

As shown in Figure S24, initially the driving moment from the nonuniform temperature of the section is larger than the friction resistance moment, the torus starts everting. Since the relative speed between the torus and the hot plate is not zero, they slide relative to each other. Because the inclined thread on the torus surface leads to anisotropic friction, there exists tangential component of the sliding friction force, which drives the torus to rotate. With the increase of angular velocity of eversion and rotation, the tangential friction decreases to zero. At the same time, the friction has only radial component, and the heat-driven driving moment equals to the friction resistance moment. The torus evolves into the steady state.

3.2 Steady eversion and rotation

As shown in Figure S24, the radius of the cross section is R, the curvature radius of the tours is ρ. The included angle between helical thread and radial direction is θ_f, The anisotropic friction coefficients of threads are μ_1 and μ_2, respectively. For the steady movement, the angular velocity of eversion is ω_E, and the angular velocity of rotation is ω_R. The relative velocity components between the contact point and the hot surface are

$$\begin{aligned} V_{r1} &= \omega_E R\cos\theta_f + \omega_R \rho \sin\theta_f , \\ V_{r2} &= \omega_E R\sin\theta_f - \omega_R \rho \cos\theta_f . \end{aligned} \qquad (1)$$

According to the anisotropic sliding friction theory [1], we can obtain

$$\frac{\omega_E R\cos\theta_f + \omega_R \rho \sin\theta_f}{\omega_E R\sin\theta_f - \omega_R \rho \cos\theta_f} = \frac{\mu_2^2}{\mu_1^2}\cot\theta_f . \qquad (2)$$

Eq. (2) gives the relationship between ω_E and ω_R,

$$\frac{\omega_R}{\omega_E} = \frac{R}{\rho} \frac{\frac{\mu_2^2}{\mu_1^2} - 1}{\tan^2 \theta_f + \frac{\mu_2^2}{\mu_1^2}} \tan \theta_f \ , \qquad (3)$$

which depends on the three dimensionless parameter $\frac{R}{\rho}$, $\frac{\mu_2}{\mu_1}$, θ_f, and is independent on the temperature.

4. References.

1. Zmitrowicz, A., 1981. A theoretical model of anisotropic dry friction. Wear, 73(1), pp.9-39.

5. Supplementary Video captions

Video S1. Self-oscillation of a single ring with different twist number.

This video shows continuous oscillations of a single ring with different twist number. The temperature of the hotplate is 85°C. The video is played with 4× accelerated speed.

Video S2. Self-oscillation of knot structure ring.

This video shows continuous oscillations of a knot structure ring. The temperature of the hotplate is 120°C. The video is played with 4× accelerated speed.

Video S3. Self-oscillation of two link rings.

This video shows continuous oscillations of two link rings. The temperature of the hotplate is 120°C. The video is played with 4× accelerated speed.

Video S4. Self-oscillation of two link rings with different rotation direction.

This video shows continuous oscillations of two link rings with different rotation direction. The temperature of the hotplate is 120°C. The video is played with 4× accelerated speed.

Video S5. Self-oscillation of three link rings with left rotation direction.

This video shows continuous oscillations of three link rings with left rotation direction. The temperature of the hotplate is 130°C. The video is played with 4× accelerated speed.

Video S6. Self-oscillation of three link rings with right rotation direction.

This video shows continuous oscillations of three link rings with right rotation direction. The temperature of the hotplate is 130°C. The video is played with 4× accelerated speed.

Video S7. Self-oscillation of three link rings with left-right-left rotation direction.

This video shows continuous oscillations of three link rings with left-right-left rotation direction. The temperature of the hotplate is 130°C. The video is played with 4× accelerated speed.

Video S8. Self-oscillation of three link rings with right-left-left rotation direction.

This video shows continuous oscillations of three link rings with right-left-left rotation direction. The temperature of the hotplate is 130°C. The video is played with 4× accelerated speed.

Video S9. Self-oscillation of three link rings with different diameter.

This video shows continuous oscillations of three link rings with different diameter. The temperature of the hotplate is 130°C. The video is played with 4× accelerated speed.

Video S10. Self-oscillation of four link rings with left rotation direction.

This video shows continuous oscillations of four link rings with left rotation direction. The temperature of the hotplate is 130°C. The video is played with 4× accelerated speed.

Video S11. Self-oscillation of five link rings with left rotation direction.

This video shows continuous oscillations of five link rings with left rotation direction. The temperature of the hotplate is 130°C. The video is played with 4× accelerated speed.

Video S12. Change three left rotation link rings direction by flipping.

This video shows continuous oscillations of three link rings with left rotation direction and change their direction by flipping. The temperature of the hotplate is 130°C. The video is played with 4× accelerated speed.
